# Generation of hypoimmunogenic universal iPS cells through HLA-type gene knockout

**DOI:** 10.1038/s12276-025-01422-3

**Published:** 2025-03-14

**Authors:** Juryun Kim, Yoojun Nam, Doyeong Jeon, Yujin Choi, SeonJu Choi, Chang Pyo Hong, Siyoung Kim, Hyerin Jung, Narae Park, Yeowon Sohn, Yeri Alice Rim, Ji Hyeon Ju

**Affiliations:** 1 YiPSCELL Inc., Seoul, Republic of Korea; 2 Department of Biohealth Regulatory Science, Sungkyunkwan University, Suwon, Republic of Korea; 3 CiSTEM Laboratory, Convergent Research Consortium for Immunologic Disease, Seoul St. Mary’s Hospital, College of Medicine, The Catholic University of Korea, Seoul, Republic of Korea; 4 Division of Rheumatology, Department of Internal Medicine, Seoul St. Mary’s Hospital, Institute of Medical Science, College of Medicine, The Catholic University of Korea, Seoul, Republic of Korea; 5 Department of Biomedicine and Health Sciences, Seoul St. Mary’s Hospital, College of Medicine, The Catholic University of Korea, Seoul, Republic of Korea

**Keywords:** Induced pluripotent stem cells, Genetic engineering, Stem cells

## Abstract

Hypoimmunogenic universal induced pluripotent stemn (iPS) cells were generated through the targeted disruption of key genes, including human leukocyte antigen (*HLA*)-*A*, *HLA-B* and *HLA-DR alpha* (*DRA*), using the CRISPR–Cas9 system. This approach aimed to minimize immune recognition and enhance the potential of iPS cells for allogeneic therapy. Heterozygous iPS cells were used for guide RNA design and validation to facilitate the knockout (KO) of the *HLA-A*, *HLA-B* and *HLA-DRA* genes. The electroporation of iPS cells using the selected guide RNAs enabled the generation of triple-KO iPS cells, followed by single-cell cloning for clone selection. Clone A7, an iPS cell with targeted KOs of the *HLA-A*, *HLA-B* and *HLA-DRA* genes, was identified as the final candidate. Messenger RNA analysis revealed robust expression of pluripotency markers, such as octamer-binding transcription factor 4, sex-determining region Y box 2, Krüppel-like factor 4, Lin-28 homolog A and Nanog homeobox, while protein expression assays confirmed the presence of octamer-binding transcription factor 4, stage-specific embryonic antigen 4, Nanog homeobox and tumor rejection antigen 1–60. A karyotype examination revealed no anomalies, and three-germ layer differentiation assays confirmed the differentiation potential. After interferon gamma stimulation, the gene-corrected clone A7 lacked HLA-A, HLA-B and HLA-DR protein expression. Immunogenicity testing further confirmed the hypoimmunogenicity of clone A7, which was evidenced by the absence of proliferation in central memory T cells and effector memory T cells. In conclusion, clone A7, a triple-KO iPS cell clone that demonstrates immune evasion properties, retained its intrinsic iPS cell characteristics and exhibited no immunogenicity.

## Hypoimmunogenic iPS cells generated for enhanced allogeneic therapy

This study focuses on improving cell therapy by reducing the immune response to induced pluripotent stem (iPS) cells. Researchers aimed to generate iPS cells that are less likely to be rejected by the immune system by editing specific genes. The team used a tool called CRISPR–Cas9 to remove certain genes, known as HLA-A, HLA-B and HLA-DRA, from iPS cells. By removing these genes, researchers hoped to make the iPS cells less recognizable to the immune system. The study found that the edited iPS cells did not trigger a strong immune response in lab tests. The edited cells maintained their ability to differentiate into various cell types and showed reduced signs of being attacked by immune cells. This research suggests that editing specific genes in iPS cells can make them more suitable for use in therapies without being rejected by the body.

This summary was initially drafted using artificial intelligence, then revised and fact-checked by the author.

## Introduction

In cell therapy, overcoming immunogenicity is crucial for the successful application of induced pluripotent stem (iPS) cells. Research utilizing adult stem cells, such as mesenchymal stem cells, embryonic stem cells and iPS cells, has been advancing^1^. iPS cells, which are generated by introducing Yamanaka factors into somatic cells, possess pluripotent characteristics and the ability to differentiate into various cell types, making them promising candidates for therapeutic use^2,3^. However, allogeneic therapy using iPS cells is challenging owing to immunogenicity^4^. To address this issue, we aim to generate hypoimmunogenic universal iPS cells by editing human leukocyte antigen (*HLA*) genes.

The HLA system is crucial for transplantation^5^. Although HLA polymorphisms are imperative for immune defense, they often cause failure owing to immune reactions as a result of genetic disparities in organ transplantation^6^. This phenomenon extends to cell therapies, where mismatched HLA types from the donor are identified as nonself, resulting in potential attacks by CD4 T cells, CD8 T cells and natural killer (NK) cells, thereby triggering an ensuing immune response^7^.

The major histocompatibility complex (MHC), also known as *HLA*, contains genes responsible for encoding the MHC molecules that function within the immune system^8,9^. Located on chromosome 6, it consists of HLA class I (A, B and C) and class II (DR, DQ and DP) molecules, representing the most polymorphic regions within the human genome. Class I MHC molecules, such as HLA-A, B and C, present foreign proteins to cytotoxic T cells in the presence of nonself proteins. They consist of a polymorphic α-chain and a nonpolymorphic β2M chain. Class II MHC molecules, including *HLA-DR*, *HLA-DQ* and *HLA-DP*, consist of a polymorphic β1 chain, a nonpolymorphic α chain and β2. They present foreign proteins to helper T cells upon the detection of nonself proteins^10,11^.

Although patient–donor HLA typing alignment is optimal for transplantation, identifying such matches poses challenges^12^. In cases of partial HLA mismatch, the degree of mismatch can notably influence the severity of immune rejection reactions. HLA matching is crucial in transplantation^13^. However, despite the existence of a bone marrow registry with four million donors in the USA, only 50–60% of cases find matches for HLA-A and HLA-B. Autologous transplantation is the most desirable method^14,15^, but it involves substantial time and expense before transplantation. Allogeneic transplantation using iPS cells from donors with homozygous HLA alleles can broaden their applicability to a more diverse patient population. However, covering all HLA types within a population is impractical. In Korea, there are examples of 13 good manufacturing practice (GMP)-grade homozygous iPS cells obtained by assessing common homozygous HLA-A, HLA-B and DRB1 types^16^, but covering the entire Korean population is still challenging. Similarly, in Japan, where 140 homozygote iPS cells can cover 90% of the population^17^, identifying such homozygous iPS cells remains a important challenge.

Researchers are exploring the use of iPS cells in transplantation by correcting mutations or editing *HLA* using gene-editing techniques, such as CRISPR–Cas9. These genetically modified iPS cells can differentiate into target cells and be administered to patients. Currently, studies are focused on generating hypoimmunogenic iPS cells using gene-editing methods for transplantation.

The CRISPR–Cas9 system is used for gene editing by targeting specific gene regions to induce DNA breaks. This facilitates nonhomologous end joining to introduce random mutations or homology-directed repair to insert the desired genes^18^. When a transfection-delivered guide RNA (gRNA) binds to the target sequence, the Cas9 enzyme precisely cuts the intended region, enabling sequence editing. This technique can be used to create knockouts (KOs) of polymorphic *HLA* genes and eliminate genes that trigger immune responses. Since its inception in 2012, CRISPR–Cas9 has been widely used across various fields, including in the development of gene and cell therapies^19^. The clinical trial registration system (http://clinicaltrials.gov) identified 49 clinical trials that used this technology, with one clinical approval for applying this technology to iPS cells^20^.

Numerous studies on *HLA* gene engineering of existing iPS cells are in progress^21–23^, aiming to establish clinical-grade universal iPS cells within GMP facilities^24^.

Recent studies have used CRISPR–Cas9 technology to induce the KO of *HLA* genes^25^. Studies on universal iPS cells have involved deleting *HLA-A*, *HLA-B*, *HLA-C*, *β2M*^26–28^ or class II MHC transactivator (*CIITA*) to eliminate MHC classes I and II^11,29,30^. Without gene editing, these cells are susceptible to cytotoxic T cells, helper T cells and NK cells. When MHC class I is completely deleted because of β2M, cytotoxic T and helper T rejection can be prevented, but NK cell attacks cannot be avoided. HLA-E blocks NK and CD8^+^ T cell attacks^31^, whereas HLA-G blocks attacks from CD8^+^ T, CD4^+^ T, B (ref. ^32^) and NK cells and macrophages, prompting research into selective deletion methods. In addition, strategies involving knocking in CD47 (ref. ^11^), which interacts with SIRPα to signal ‘don’t-eat me’^33^, can evade recognition and removal by macrophages^34,35^. Selective KO methods include biallelic KO of *HLA-A* and *HLA-B* genes, monoallelic KO of the HLA-C gene and KO of *CIITA* to eliminate all HLA class II molecules^36,37^.

This study aims to generate hypoimmunogenic iPS cells by eliminating immunogenicity through the deletion of the *HLA-A, HLA-B* and *HLA-DRA* genes, which are closely associated with recipient T cell responses among donor HLA types during cell transplantation. Using the CRISPR–Cas9 system, the *HLA-A*, *HLA-B* and *HLA-DRA* genes were deleted in the YiP3 cell line, which is derived from peripheral blood mononuclear cell (PBMC)-derived iPS cells with heterozygosity for the *HLA* gene, to prevent immune rejection reactions. Previous studies on universal iPS cells have used homozygous iPS cells or iPS cells with partially matching alleles in the *HLA* genes as gene-editing targets^38^. By contrast, this study aims to genetically modify both alleles in heterozygous iPS cells. The majority of existing studies and patents have focused on deleting *HLA-A*, *HLA-B* and *HLA-DRB1* (ref. ^39^), with no research reporting on knocking out *HLA-DRA*. This strategy involves deleting the DRA α-chain, thereby eliminating the structure of MHC class II, which is responsible for antigen recognition. With respect to the *HLA-DR* component, considering the presence of *HLA-DRA* alongside various HLA-DRB genes, such as *HLA-DRB1*, *HLA-DRB3*, *HLA-DRB4* and *HLA-DRB5*, knocking out only DRB1 could result in the continued expression of DRB3, DRB4 and DRB5. To address this concern, the strategy of knocking out HLA-DRA was selected. In this study, we established hypoimmunogenic iPS cells to mitigate immune responses, which are potential adverse effects of allogeneic transplantation. Quality testing and immunogenicity assessments of the iPS cells were conducted to prepare them for use in cell therapy.

## Materials and methods

### Gene-edited iPS cells

On day 0, CRISPR–Cas9 reagents (ribonucleoprotein (RNP) complexes) were prepared by combining 80 µg of gRNA for *HLA-A*, *HLA-B* and *HLA-DRA* with 4 µg/µl CAS9 (Alt-R Sp Cas9 Nuclease V3, IDT). Subsequently, 1 million PBMC-derived iPS cells (YiP3) were mixed with these complexes and subjected to electroporation. Electroporation was performed using a Lonza 4D-Nucleofector X Unit with the program code CA-137. From days 3–5, the transfected cells were collected and subjected to single-cell cloning using flow cytometry. Approximately 10,000 cells were collected for electroporation pool analysis. The transfected electroporation pool was genotyped from days 5–13. After the transfected iPS cells were seeded into a 96-well plate, they were cultured with mTeSR-Plus, and single clones were selected. From days 11–13 to days 25–28, the gene-edited clones were analyzed by PCR and Sanger sequencing.

### iPS cell culture

The gene-edited cell lines were thawed and passaged following the Supplementary Methods. After the cells stabilized, the coating material and culture medium were replaced to culture the cells under conditions similar to those of the control iPS cells. The detailed methods are described in the supplementary methods.

### EC differentiation

Endothelial cells (ECs) were generated as previously described^40^. The detailed methods used for differentiating ECs from iPS cells are described in the supplementary methods.

### Alkaline phosphatase staining

Alkaline phosphatase staining was performed using an alkaline phosphatase detection kit (SCR004; Sigma) following the manufacturer’s protocol. The detailed protocol is described in the supplementary methods.

### Flow cytometry

The detailed methods and antibodies used for flow cytometry are provided in the Supplementary Methods.

### Three-germ lineage differentiation assay

The differentiation potential of the trilineage germ cell layers was determined using a STEMdiff Trilineage Differentiation Kit (05230; STEMCELL Technology) following the manufacturer’s protocol. The detailed protocol is described in the Supplementary Methods.

### Quantitative real-time PCR

Total RNA was isolated using TRIzol reagent (15596026; Invitrogen). Complementary DNA was synthesized from the isolated RNA using a RevertAid First Strand cDNA Synthesis Kit (K1622; Thermo Fisher) following the manufacturer’s protocol. Quantitative real-time PCR was performed using a QuantStudio 3 instrument (Applied Biosystems) and Power SYBR Green PCR Master Mix (4367659; Applied Biosystems). The primer details are listed in Supplementary Table 1.

### Sanger sequencing

After genomic DNA extraction from iPS cells, PCR and Sanger sequencing were performed using the primers listed in Supplementary Tables 2–4.

### Immunofluorescence assay

The detailed methods and antibodies used are provided in the Supplementary Methods.

### Western blotting

The detailed methods and antibodies used for immunoblotting are described in the Supplementary Methods.

### In vitro immunogenicity assay

An in vitro immunogenicity assay was performed to assess the CD4^+^ T cell response. The detailed methods are described in the Supplementary Methods.

### Assessment of genetic stability

#### CNV analysis

The genomic DNAs of YiP3 iPS cells (wild type) and HLA-triple-KO clones (A7 and B2) were genotyped using a CytoScan HD array (Thermo Fisher Scientific) following the manufacturer’s protocol. The data analysis was conducted using the Chromosome Analysis Suite (version 4.4.0.63; Thermo Fisher Scientific). The detailed methods are described in the Supplementary Methods.

### WGS and bioinformatic analysis

Sequencing libraries of YiP3, A7 and B2 were prepared from input DNA (1 µg) using a TruSeq DNA sample prep kit (Illumina) following the manufacturer’s protocols. These libraries were subjected to paired-end sequencing using a 150-bp read length on the Illumina NovaSeq 6000 platform (Illumina). The detailed methods are described in the Supplementary Methods.

### RNA sequencing and bioinformatic analysis

cDNA libraries were prepared from the total RNA (1 μg) of each sample using a TruSeq Stranded mRNA Sample Prep Kit (Illumina) following the manufacturer’s protocols. After qPCR validation, the libraries were subjected to paired-end sequencing with a 150-bp read length on the Illumina NovaSeq 6000 platform (Illumina). The detailed methods are described in the Supplementary Methods.

### Statistical analysis

Statistical analyses were conducted using GraphPad Prism software (v. 5.01; GraphPad). Statistical significance was assessed using a two-way analysis of variance or Student’s *t*-test and is expressed as follows: *P < 0.05, **P < 0.01 ***P < 0.001.

## Results

### Design of triple-*HLA-*gene-KO iPS cells

**Fig. 1:**
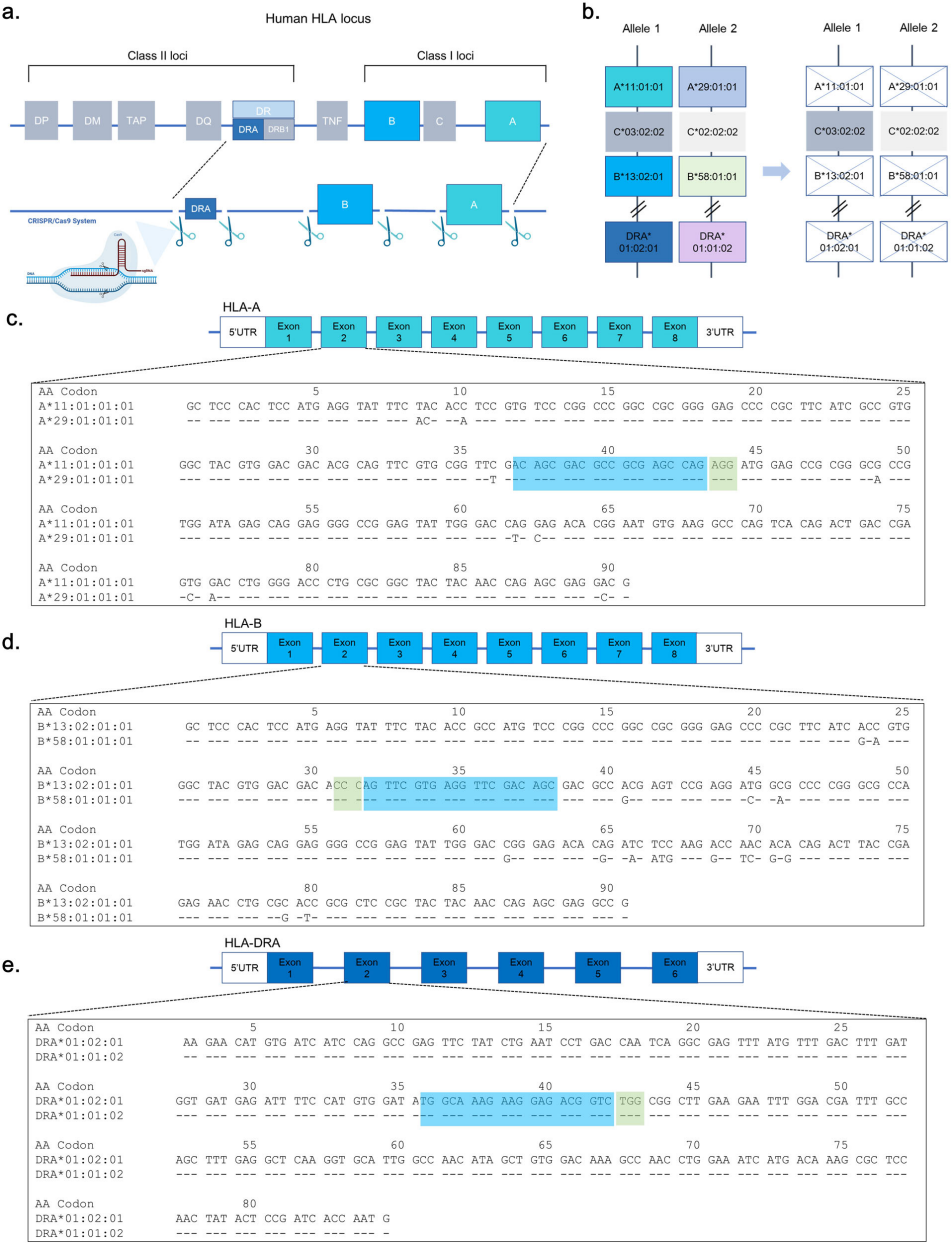
Design of triple-*HLA*-gene-KO iPS cells. **a**, A scheme of triple-*HLA*-gene-KO iPS cells. **b**, A strategy scheme of the HLA type of YiP3 iPS cells. **c**–**e**, Exon 2 nucleotide sequences of *HLA-A* (**c**), *HLA-B* (**d**) and *HLA-DRA* (**e**) for YiP3 iPS cells are displayed in codons. The blue part of the sequence represents the gRNA region, and the green part represents the protospacer adjacent motif (PAM) site. The nucleotide sequence is displayed using the sequence alignment tool in the immune polymorphism database-international immunogenetics information system/human leukocyte antigen (IPD-IMGT/HLA) database.

To generate hypoimmunogenic iPS cells without immune rejection, we used YiP3 PBMC-derived iPS cells with heterozygous alleles for the *HLA* genes on chromosome 6. The YiP3 line carried the following alleles: *HLA-A* 11:01:01:01 and *HLA-A* 29:01:01:01, *HLA-B* 13:02:01:01 and *HLA-B* 58:01:01:01, *HLA-C* 03:02:02 and *HLA-C* 02:02:02, and *HLA-DRA* 01:02:01 and *HLA-DRA* 01:01:02. Our strategy aimed to KO *HLA-A* and *HLA-B*, which represent polymorphisms in class I of the HLA locus, using CRISPR–Cas9 and KO *HLA-DRA* to prevent the expression of *HLA-DR*, which represents a polymorphism in class II, while leaving *HLA-C*, which has a minor polymorphism (Fig. 1a, b). The CRISPR–Cas9 system was used for gene KO. For each gene, two gRNAs were designed to target regions excluding heterogeneous regions within the *HLA-A* gene, where a protospacer adjacent motif (PAM) site (NGG) exists and may act bialleically on a 20-bp sequence (Supplementary Fig. 1a–c). For example, using the immuno polymorphism database-international immunogenetics information system (IPD-IMGT)/HLA database^41^, each allele was aligned. We designed a gRNA (G0002-HLA-A-g1, ACAGCGACGCCGCGAGCCAG, PAM:AGG) that targets codons 37–43 within exon 2 for *HLA-A*. We designed a gRNA (G0002-HLA-B-g1, GCTGTCGAACCTCACGAACT, PAM:GGG) that targets codons 31–38 within exon 2 for *HLA-B* and a gRNA (G0002-HLA-DRA-g2, TGGCAAAGAAGGAGACGGTC, PAM:TGG) that targets codons 36–42 within exon 2 for *HLA-DRA* (Fig. 1c–e).

### Assessment of the designed gRNAs

**Fig. 2:**
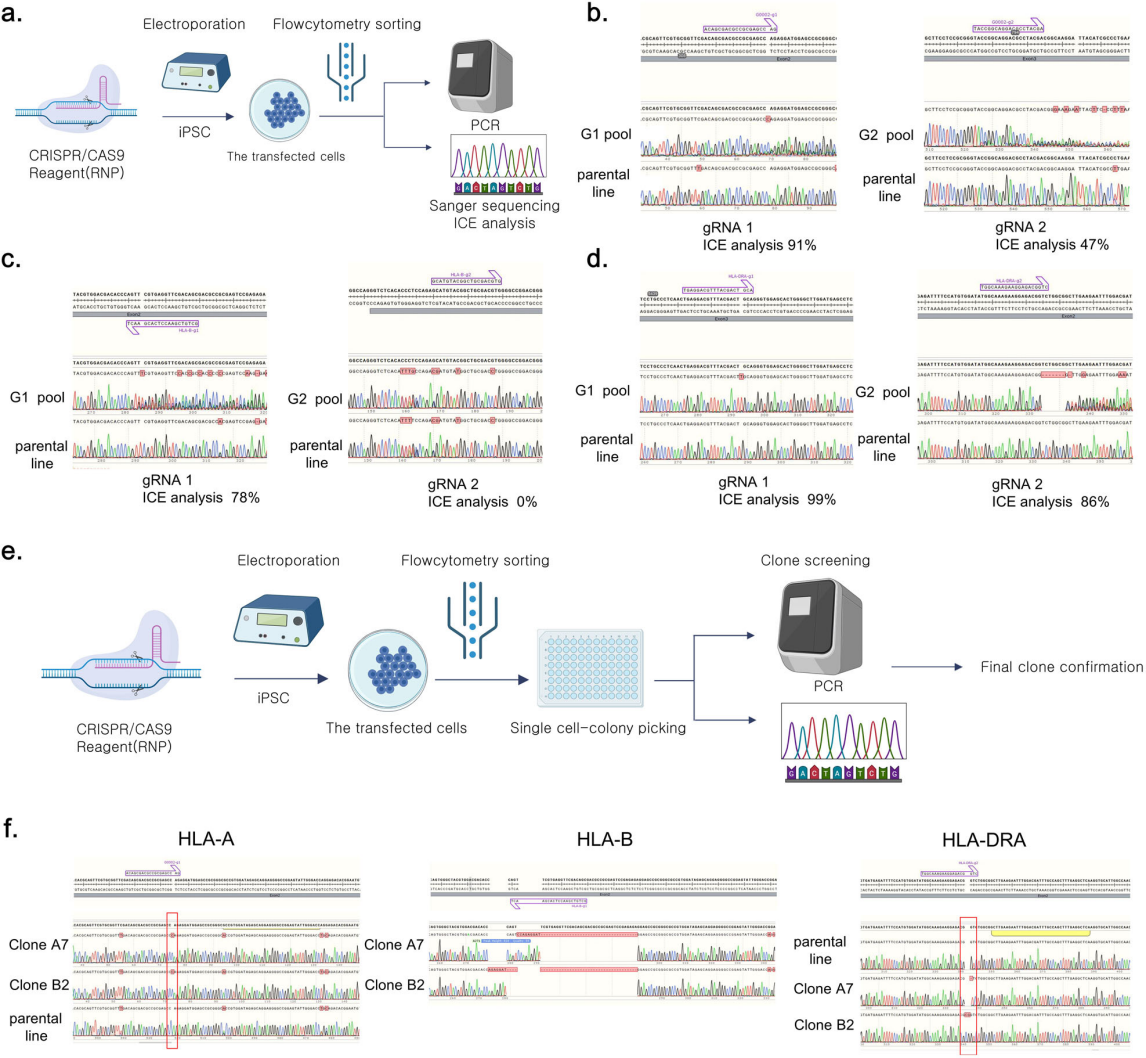
Assessment of gRNAs in iPS cells and confirmation of triple-*HLA*-gene-KO iPS cells. **a**, A scheme of gRNA assessment in iPS cells. This illustration was created using bioRender. **b**, The Sanger sequence images and inference of CRISPR edits (ICE) analysis demonstrating the efficiency of two candidate gRNAs per *HLA-A* gene. **c**, The Sanger sequencing images and ICE analysis demonstrating the efficiency of two candidate gRNAs per *HLA-B* gene. **d**, Sanger sequence images and ICE analysis demonstrating the efficiency of two candidate gRNAs per *HLA-DRA* gene. **e**, A schematic representation of the single-clone selection process for iPS cells in which *HLA-A*, *HLA-B* and *HLA-DRA* were knocked out. This illustration was created using bioRender. **f**, The Sanger sequencing images demonstrating the KO of the *HLA-A*, *HLA-B* and *HLA-DRA* genes in the YiP3 iPS cell line. A7 and B2 represent KO clones, with each clone demonstrating the KO of the *HLA-A* and *HLA-B* genes, respectively, while the *HLA-DRA* gene is also knocked out.

The efficiency of CRISPR–Cas9 gRNAs designed for the *HLA-A*, *HLA-B* and *HLA-DRA* genes was assessed by electroporating bulk iPS cells with each gRNA and measuring the transfection efficiency (Fig. 2a). Sanger sequencing and inference of CRISPR edits (ICE) analysis of the transfected iPS cell pool revealed that, for *HLA-A* gRNA, G0002-HLA-A-g1 and G0002-HLA-A-g2 demonstrated 91% and 47% efficiency, respectively. Therefore, G0002-HLA-A-g1 was selected as the final gRNA. For *HLA-B* gRNA, G0002-HLA-B-g1 and G0002-HLA-B-g2 demonstrated 78% and 0% efficiency, respectively, indicating a lack of match. Therefore, G0002-*HLA-B*-g1 was selected as the final gRNA. For *HLA-DRA*, G0002-HLA-DRA-g1 and G0002-HLA-DRA-g2 demonstrated 99% and 86% efficiency, respectively. However, considering that the *HLA-DR* polymorphism predominantly occurs within exon 2, G0002-HLA-DRA-g1, which targets this region, was selected as the final gRNA (Fig. 2b–d).

### Confirmation of triple-*HLA*-gene-KO iPS cells

To obtain engineered YiP3 cells with triple-*HLA*-gene KOs, single clones were obtained through electroporation-mediated transfection using selected gRNAs that target the *HLA-A*, *HLA-B* and *HLA-DRA* genes (Fig. 2e). To establish the concentration conditions for each gRNA for transfection, 40 and 80 µg of each gRNA were used to form the RNP complex. The efficiencies of the KO scores for each gene, determined through ICE analysis, were 53%, 37%, 44%, 58%, 45% and 56%. Subsequently, transfection was performed using 80 µg of each gRNA to generate the RNP complex (Supplementary Fig. 2a). Single-cell cloning was conducted on the transfected iPS cells, followed by electroporated pool (EP pool) analysis and genotyping using next-generation sequencing (NGS). Subsequently, 48 clones were collected by seeding them into a 96-well plate, and each clone was screened and genotyped using Sanger sequencing. Six potential triple-KO clones were selected and further assessed through confirmatory sequencing and/or NGS (Supplementary Fig. 2b).

Clone B4 had mixed alleles in the *HLA-B* region, whereas clone C6 had unresolved issues in the *HLA-A* region and was excluded from further assays (Supplementary Fig. 2b). The genotyping results for the triple-KO clones were further verified by sequencing for final confirmation. Clone A7 exhibited homozygosity with a 1-bp insertion, 28-bp deletion and 1-bp deletion in the *HLA-A*, *HLA-B* and *HLA-DRA* regions, respectively. Clone B2 exhibited homozygosity with a 1-bp insertion, 34-bp deletion and 2-bp insertion in the *HLA-A*, *HLA-B* and *HLA-DRA* regions, respectively (Fig. 2f and Supplementary Fig. 2b). Moreover, clones B3 and B11 presented incomplete *HLA-A* regions with a 1-bp insertion and a 1-bp deletion near g1 (Supplementary Fig. 2b, c). Therefore, based on the genotyping results, clones A7 and B2 were selected as strong candidates for triple-*HLA-*gene KO, and further comparative analysis was conducted along with clones B3 and B11 through additional assays.

### Engineered triple-KO iPS cells retain pluripotency

**Fig. 3:**
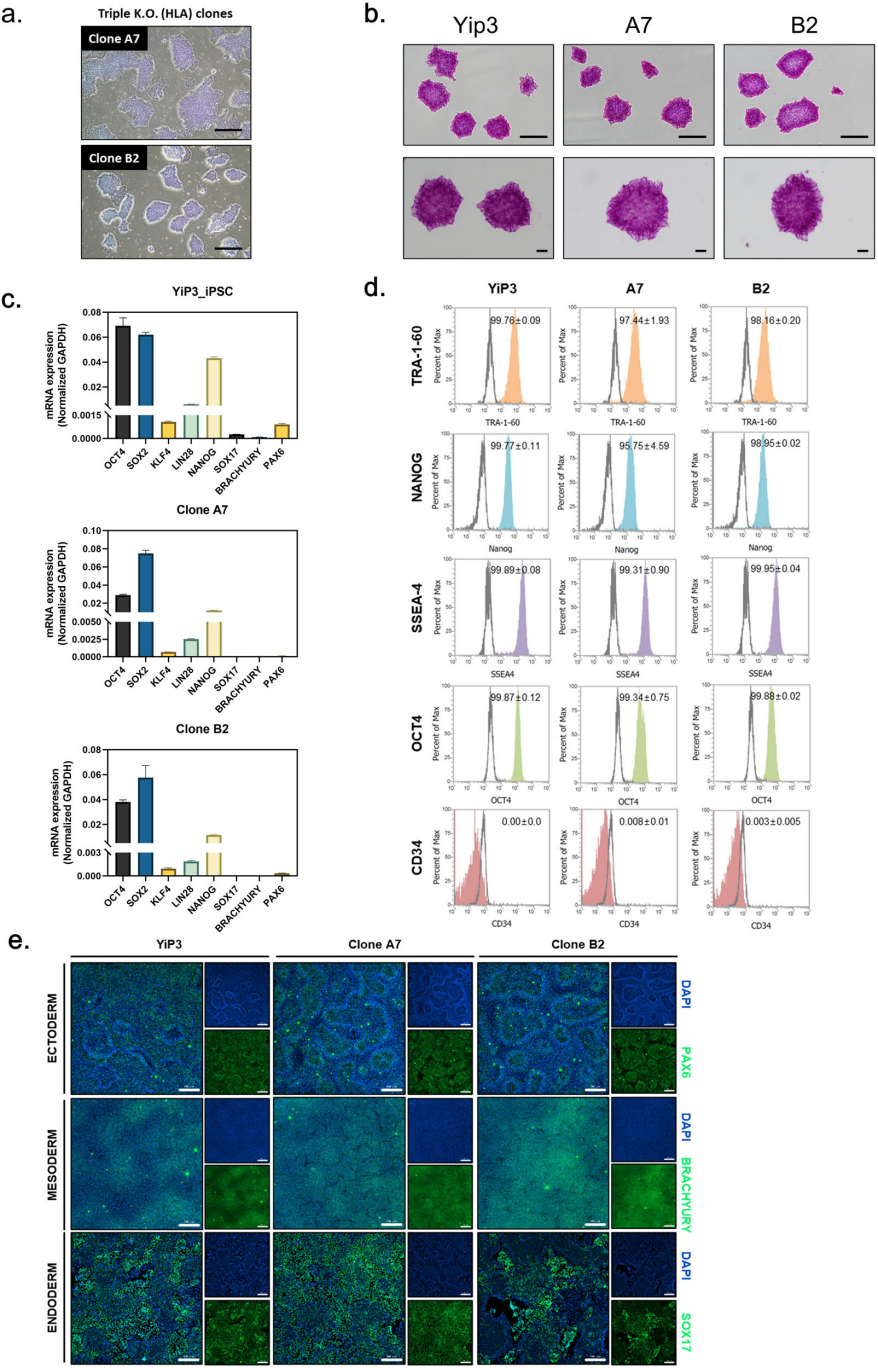
Assessment of the characteristics of engineered iPS cells. **a**, A microscopy image of iPS cells and clones A7 and B2 with *HLA-A*, *HLA-B* and *HLA-DRA* knocked out. Scale bars, 500 μm. **b**, Alkaline phosphatase (AP) staining image of iPS cells, YiP3 and clones A7 and B2. Scale bars, 500 μm. **c**, Real-time PCR data for the pluripotency markers, such as *OCT4*, *SOX2*, *KLF4*, *LIN28* and *NANOG*, and three-germ layer differentiation markers, such as *SOX17*, *BRACHYURY* and *PAX6*, in YiP3 and clones A7 and B2. **d**, Flow cytometry data for pluripotency markers, such as tumor rejection antigen 1-60 (TRA-1-60), NANOG, stage-specific embryonic antigen 1 (SSEA4), OCT4 and the negative marker CD34 in YiP3 and clones A7 and B2. **e**, The immunocytochemistry images demonstrating the expression of markers (PAX6) for ectoderm, (BRACHYURY) for mesoderm and (SOX17) for endoderm, and 4',6-diamidino-2-phenylindole (DAPI) for nuclear staining following differentiation into the three germ layers using YiP3 and clones A7 and B2. Scale bars, 200 μm.

To assess pluripotency, quality testing was conducted on triple-KO iPS cell clones in comparison with the control YiP3 cells. Morphologically, clones A7 and B2 exhibited colony formation (Fig. 3a), and positive staining for alkaline phosphatase confirmed their undifferentiated state (Fig. 3b). At the mRNA level, the expression of pluripotency markers, such as octamer-binding transcription factor 4 (*OCT4*), sex-determining region Y (SRY)-box 2 (*SOX2*), Krüppel-like factor 4 (*KLF4*), Lin-28 homolog A (*LIN28*) and Nanog homeobox (*NANOG*), was confirmed, whereas the endoderm differentiation marker (*SOX17*), mesoderm differentiation marker (*BRACHYURY*) and ectoderm marker (paired box 6, *PAX6*) were not expressed (Fig. 3c). Similar patterns were observed in clones B3 and B11 (Supplementary Fig. 3a–c).

To compare the expression of pluripotency markers at the protein level using preengineered YiP3 cells, flow cytometry was subsequently performed for OCT4, stage-specific embryonic antigen 4 (SSEA4), NANOG, tumor rejection antigen 1-60 (TRA-1-60) and the negative marker (CD34). The results demonstrated that clones A7 and B2 expressed OCT4, SSEA4, NANOG and TRA-1-60 at levels exceeding 95% in cell populations, comparable with YiP3 cells (Fig. 3d). Clone B11 presented expression levels exceeding 99% compared with those of YiP3 cells, whereas clone B3 presented a lower expression of NANOG at 86% (Supplementary Fig. 3d).

To assess the ability of each clone to differentiate into the three-germ layers (endoderm, mesoderm and ectoderm), lineage differentiation was induced and immunofluorescence staining for SOX17, BRACHYURY and PAX6 markers was performed. The expression of these markers was confirmed in all the clones, indicating that engineered iPS cell clones A7 and B2 retained their differentiation capacity similar to that of YiP3 without any alterations (Fig. 3e). Similar results were observed for clones B3 and B11, indicating no effect on the differentiation of the three-germ layers (Supplementary Fig. 3f).

In conclusion, among the selected triple-KO clones, clones A7 and B2 presented normal iPS cell properties in terms of pluripotency and the ability to differentiate into three-germ layers, similar to YiP3 cells. However, clone B3 was excluded because of its significantly lower NANOG expression at the protein level.

### Assessment of HLA expression

**Fig. 4:**
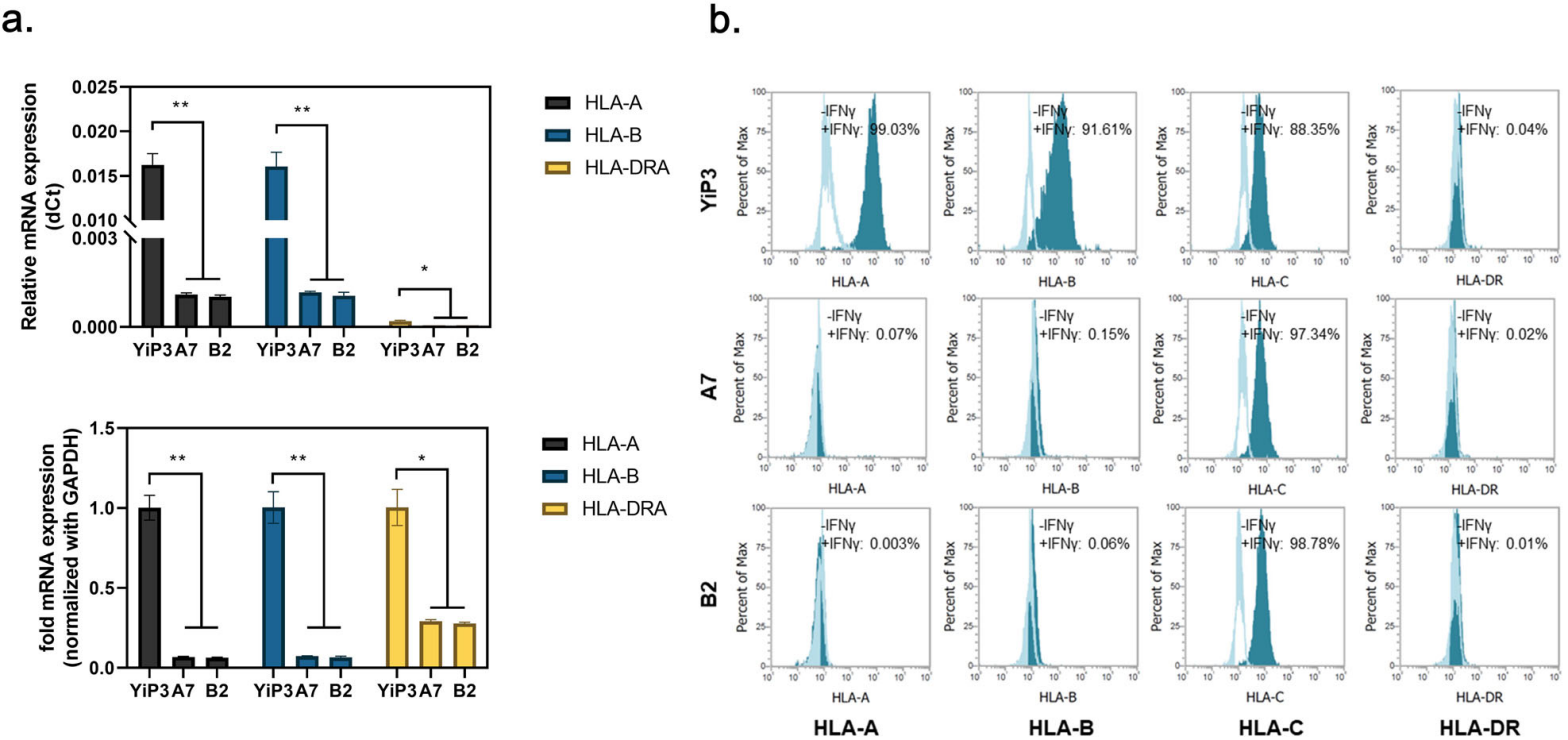
Assessment of *HLA* gene and protein expression. **a**, Real-time PCR data showing the mRNA expression levels of *HLA-A*, *HLA-B* and *HLA-DRA* in YiP3 and clones A7 and B2. Top: the relative mRNA expression in delta cycle threshold (dCt) values. Bottom: a graph normalized to glyceraldehyde 3-phosphate dehydrogenase (GAPDH), with YiP3 used as the reference value. The statistical significance is indicated by the *P* value (analysis of variance (ANOVA) test) versus YiP3: *P < 0.05, **P < 0.01 and ***P < 0.001. **b**, The flow cytometry data demonstrate alterations in the expression levels of HLA-A, HLA-B and HLA-DR in YiP3 and clones A7 and B2 iPS cells before and after stimulation with IFN-γ. The histograms represented by the lines depict the cell populations before IFN-γ stimulation, whereas those shaded in cyan represent the cell populations after IFN-γ stimulation.

We assessed the mRNA expression levels of *HLA-A*, *HLA-B* and *HLA-DRA* in the genetically edited and selected clones (A7 and B2). Real-time PCR analysis revealed significant reductions in the delta cycle threshold values of *HLA-A* and *HLA-B* compared with those of YiP3 cells (*P* < 0.01 versus YiP3) and a reduction in *HLA-DRA* expression (*P* < 0.05 versus YiP3). Normalization to glyceraldehyde 3-phosphate dehydrogenase relative to its expression in YiP3 cells revealed significantly reduced mRNA levels of *HLA-A* and *HLA-B* (*P* < 0.01 versus YiP3) and a reduction in *HLA-DRA* expression (*P* < 0.05 versus YiP3) (Fig. 4a).

Flow cytometry analysis was conducted to assess whether gene editing and selection targeting HLA-A, HLA-B and DRA in clones A7 and B2 resulted in the absence of HLA-DR protein expression. Without interferon gamma (IFN-γ) stimulation, YiP3 cells and the A7 and B2 iPS cell clones did not express HLA-A, HLA-B, HLA-DR or HLA-C (unedited region). After IFN-γ stimulation for 2 days, HLA-A, HLA-B and HLA-C protein expression increased in YiP3 cells, with HLA-A, HLA-B, HLA-C and DR increasing by 99.03, 91.61, 88.35 and 0.04%, respectively (Fig. 4b). By contrast, under IFN-γ stimulation, triple-KO clone A7 exhibited significant reductions in HLA-A (0.07%), HLA-B (0.15%) and HLA-DR (0.02%) protein expression. However, HLA-C protein expression (97.34%) remained unaffected because it was not within the gene-edited region. Similar results were observed for clone B2 (Fig. 4b). These results demonstrate selective KO of the targeted HLA-A, HLA-B and DRA regions in YiP3 at the protein level, as confirmed through our analyses.

### Genetic stability of *HLA*-triple-KO iPS cells in clones A7 and B2

**Fig. 5:**
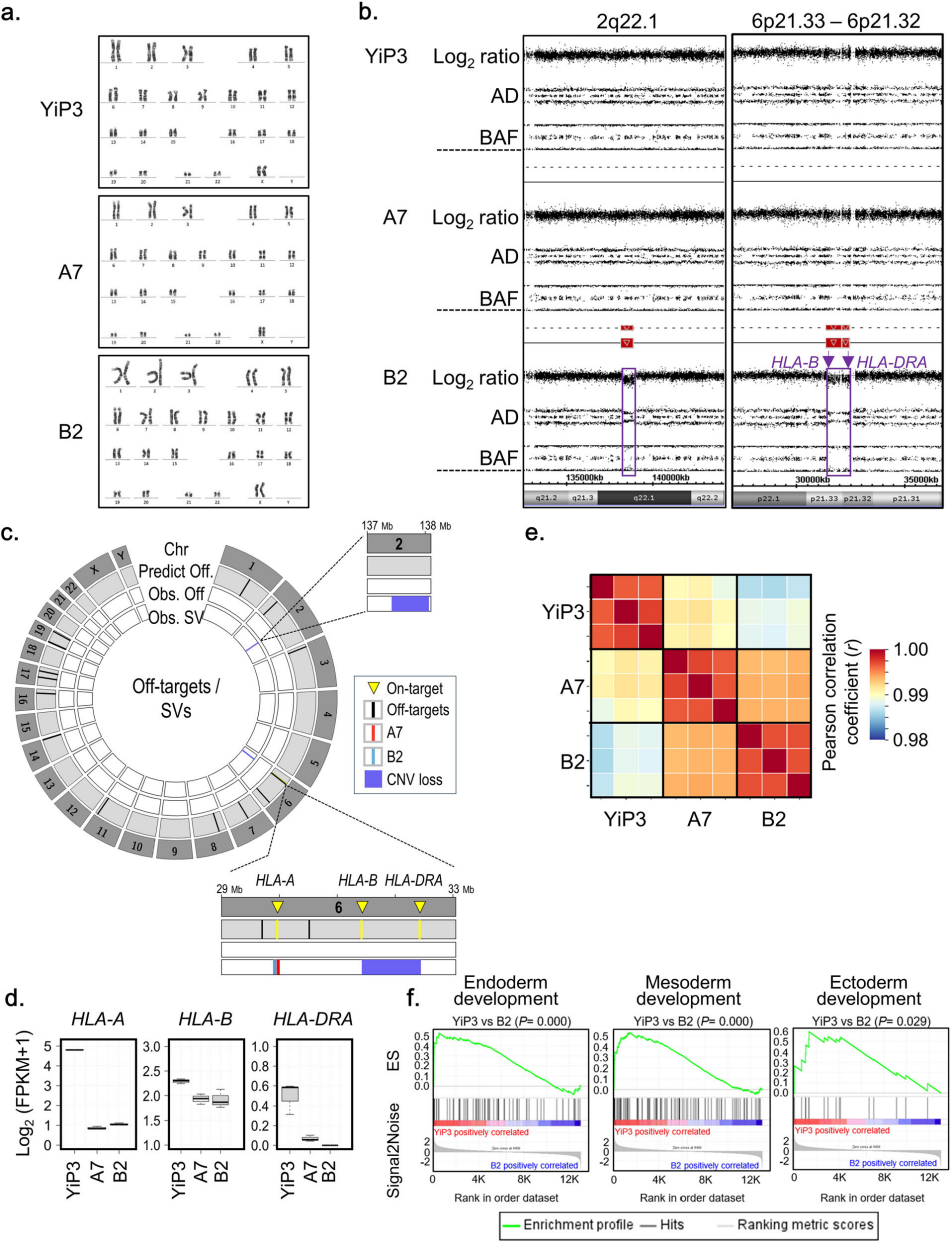
Assessment of the genetic stability of *HLA*-triple-KO iPS cells in clones A7 and B2. **a**, Karyotype analysis of YiP3 iPS cells and *HLA*-triple-KO iPS cells in clones A7 and B2. **b**, Identification of copy number (CN) losses in clone B2. CN losses at the 2q22.1 (138.17–138.82 Mbp on chromosome 2) and 6p21.33–6p21.32 (31.32–32.41 Mbp on chromosome 6) loci were identified through high-resolution single-nucleotide polymorphism (SNP) genotyping using the CytoScan HD array. AD: allele difference, BAF: B allele frequency. **c**, Identification of CRISPR–Cas9 off-target effects induced by three gRNAs in clones A7 and B2. In the Circos plot, the first layer represents all human chromosomes (Chr). The second layer represents off-target sites (Predict Off; black color bar) in the human reference genome predicted by Cas-OFFinder, facilitating two mismatches. The third layer represents off-targets (Obs. Off), such as SNVs or insertions and/or deletions (InDels), as observed from whole-genome sequence (WGS) data of clones B2 (green color bar) and A7 (red color bar). The fourth layer represents structural variants (obs. SV) related to on- and/or off-target activity detected from WGS data of clones B2 (green color bar) and A7 (red color bar). In clone A7, an SV event is detected: a 28-bp deletion in *HLA-A*. In clone B2, three SV events are detected: a 34-bp insertion in *HLA-A* and CN losses at two loci, 2q22.1 (encompassing THSD7B to HNMT) and 6p21.33–6p21.32 (encompassing *HLA-B* and *HLA-DRA*). **d**, The expression of the targeted genes *HLA-A*, *HLA-B*, and *HLA-DRA* in YiP3 and clones A7 and B2. **e**, Gene expression correlations among YiP3, A7 and B2. **f**, An enrichment plot for endoderm (GO: 0007492), mesoderm (GO: 0007498) and ectoderm (GO: 0007398) development. The plot shows the running enrichment score (ES) for the gene set (top) as the analysis continues down the ranked list (middle).

The genetic stability of *HLA*-triple-KO clones A7 and B2 was assessed by investigating karyotypes, copy number variations (CNVs), CRISPR–Cas9 off-target effects and alterations in the expression of potential cell differentiation-associated genes. Normal karyotypes were identified for clones A7 and B2 (Fig. 5a), whereas clone B11 exhibited a chromosomal abnormality with a deletion on chromosome 6p (Supplementary Fig. 3e), thereby precluding its consideration as an *HLA*-triple-KO iPS cell candidate clone. No CNV was identified in clone A7 through single-nucleotide polymorphism genotyping using the CytoScan HD array (Fig. 5b). However, copy number losses at two loci, 2q22.1 (138.17–138.82 Mbp on chromosome 2) and 6p21.33–6p21.32 (31.32–32.41 Mbp on chromosome 6), were detected (Fig. 5b). Specifically, copy number loss at 6p21.33 and 6p21.32 indicates the genomic instability of *HLA-B* and *HLA-DRA* in clone B2 (Fig. 5b). After predicting CRISPR–Cas9 off-target effects in the human reference genome using Cas-OFFinder, potential off-target Cas9 activity was detected from the whole-genome sequencing (WGS) data of clones A7 and B2. Among the 21 off-target sites predicted using Cas-OFFinder, none corresponding to off-target sites were observed in the WGS data of clones A7 and B2 (Fig. 5c). However, four structural variants that may be induced through on- and/or off-target activity were identified: a 28-bp deletion in *HLA-A* for clone A7 and a 34-bp deletion in *HLA-A* and two CNV losses for clone B2 (Fig. 5c). The structural variants identified in clones A7 and B2 were observed within coding sequences adjacent to the on-targets for *HLA-A* and *HLA-B* KO (Supplementary Table 5), indicating the possibility of additive induction in *HLA-A* and *HLA-B* KO. In addition, somatic mutations, including single-nucleotide variants (SNVs) and insertions and/or deletions, were analyzed from the whole-genome sequence data of A7 and B2 clones by comparison with YiP3 iPS cells. Somatic coding variants that may cause functional effects were not observed in clones A7 and B2 (Supplementary Table 6). In addition to assessing the genomic stability of the *HLA*-triple-KO clones, we assessed their transcriptome alterations. First, the KO genes (*HLA-A*, *HLA-B* and *HLA-DRA*) were downregulated in clones A7 and B2 compared with those in YiP3 cells (Fig. 5d), indicating the consequences of *HLA-A*, *HLA-B* and *HLA-DRA* KOs. Second, despite the KO events, strong correlations in overall gene expression were observed among YiP3, A7 and B2 cells: Pearson correlations of ≥0.99 between YiP3 and A7 cells and ≥0.98 between YiP3 and B2 cells (Fig. 5e). These results indicate that the genome-wide gene expression patterns of the *HLA*-triple-KO clones A7 and B2 closely resemble that of the wild-type YiP3 cells. Third, we also assessed the potential role of the cell differentiation process through gene set enrichment analysis. In contrast to those in clone A7, genes associated with the development of the three-germ layers (endoderm, mesoderm and ectoderm) in pluripotent stem cells were significantly downregulated in clone B2 (*P* = 0.000–0.029) (Fig. 5f). These results indicate the potential genomic instability of clone B2 owing to the downregulation of genes associated with cell development and off-target effects, such as CNV loss. Considering these results, our findings demonstrate that only the *HLA*-triple-KO clone A7 attains genomic stability.

To assess the genetic integrity of genome-edited iPS cells during long-term in vitro culture, we cultured the genome-edited clone A7 (starting at passage 15) for an additional ten passages under both normoxic (21% O_2_) and hypoxic (<5% O_2_) conditions and performed WGS and RNA sequencing (Supplementary Fig. 4a). In whole-genome sequence analysis for passage 15 and passage 25 under the two conditions, CNVs were not detected at 2q22.1 and 6p21.33-32, which were predicted to be caused by off-target effects, nor at 20q11.21, where copy number gains frequently occur during in vitro culture (Supplementary Fig. 4b). Furthermore, no sudden occurrence of somatic coding variants, including pathogenic coding variants or structural variants, was observed around the off-target sites (Supplementary Fig. 4c). These results suggest that genetic instability related to off-target effects did not occur during the long-term in vitro culture of A7. In addition to the assessment of whole-genome stability, we observed a high degree of transcriptomic similarity between passages 15 and 25 under both conditions, with strong correlations (normoxic: Pearson correlation coefficient (*r*) = 0.974; hypoxic: *r* = 0.959) (Supplementary Fig. 4d). The expression levels of the KO genes *HLA-A*, *HLA-B* and *HLA-DRA* remained highly consistent between the early and later passages, indicating stable expression of the KO genes in A7 (Supplementary Fig. 4e). The functional annotation of the DEGs revealed no notable changes in functional pathways during long-term in vitro culture under both conditions, except for changes in the expression levels of genes related to the cellular response to oxidative stress under hypoxic conditions (Supplementary Fig. 4f,g). These results demonstrate that the genome-edited clone A7 maintains transcriptomic stability during long-term in vitro culture.

### In vitro immunogenicity test

**Fig. 6:**
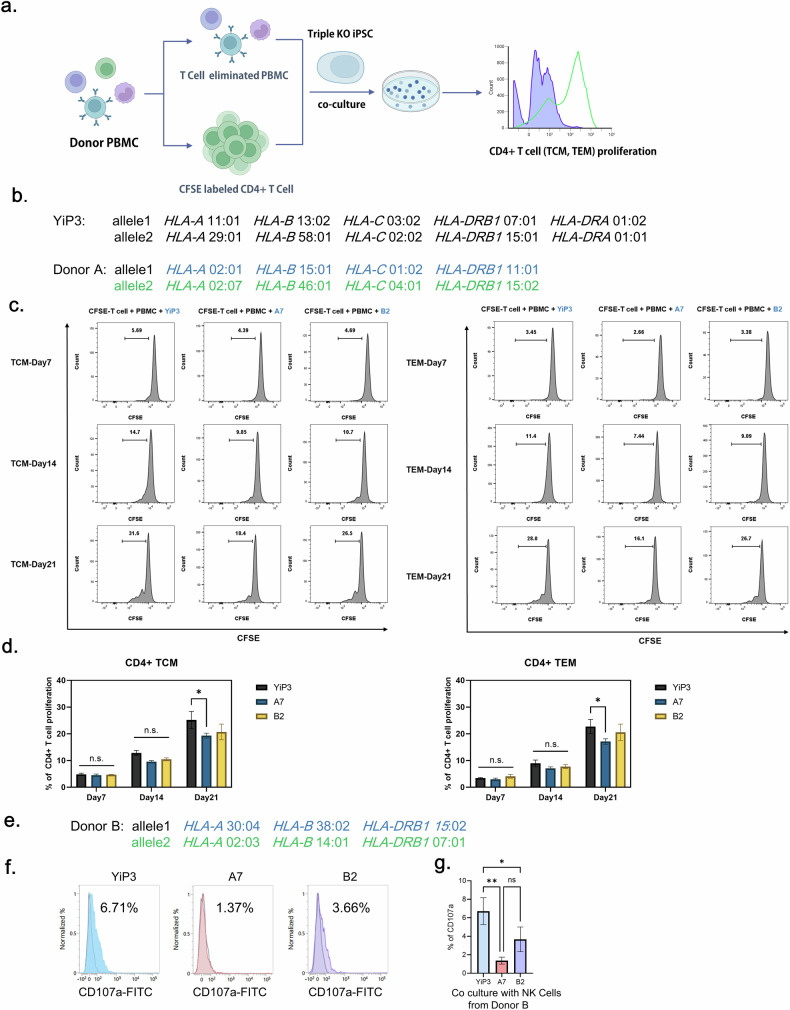
Immunogenicity test for triple-KO engineered iPS cells under IFN-γ stimulation. **a**, A schematic representation of the process of the in vitro immunogenicity test for YiP3 and clones A7 and B2. This illustration was created using bioRender. **b**, The HLA type of YiP3 and the HLA type of PBMC donor used in the immunogenicity test for YiP3 differ from the HLA type of YiP3. **c**, The histograms from flow cytometry depict the proliferation of CFSE-stained T cells and T cell-depleted donor PBMCs cocultured with YiP3 and clones A7 and B2. CFSE-labeled T cells represent TCMs (left), whereas CFSE-labeled T cells (right) represent TEMs. The proliferation levels of TCMs and TEMs were measured on days 7, 14 and 21. **d**, The data on CD4^+^ T cell proliferation percentages obtained from flow cytometry. Left: the extent of proliferation of CD4^+^ TCMs from YiP3 and clones A7 and B2 on days 7, 14 and 21. Right: the extent of proliferation of CD4^+^ TCM from YiP3 and clones A7 and B2 on days 7, 14 and 21. The statistical significance is indicated by the *P* value (analysis of variance (ANOVA) test) versus YiP3: *P < 0.05, **P < 0.01 and ***P < 0.001. **e**, The HLA type of NK cell donor used in the NK cell activity test for YiP3. **f**, A CD107a assay to detect CD107a-positive NK cells reacting to the YiP3, A7 and B2 iPS cell lines. CD107a expression was measured by flow cytometry after incubating YiP3, A7 and B2 cells with NK cells isolated from donor B. **g**, The quantification data of CD107a^+^ NK cells obtained by flow cytometry. The statistical significance is indicated by the *P* value (ANOVA) test) versus YiP3: *P < 0.05 and **P < 0.01.

To assess whether gene-edited iPS cells exhibit immunogenicity, we conducted coculture experiments using PBMCs from a donor whose HLA types differed from those of YiP3 cells. We isolated effector memory T cell (TEM) and central memory T cells (TCM) cells and assessed their proliferation levels (Fig. 6a). Initially, we selected a donor A with HLA types different from those of YiP3 cells for each allele. Allele 1 carried *HLA-A* 02:01, *HLA-B* 15:01, *HLA-C* 01:02 and *HLA-DRB1* 11:01, and allele 2 carried *HLA-A* 02:07, *HLA-B* 46:01, *HLA-C* 04:01 and *HLA-DRB1* 15:02 (Fig. 6b). Before being cocultured with PBMCs, both YiP3 cells and clones A7 and B2 were stimulated with IFN-γ for 2 days. We analyzed the proliferation of activated T cells in response to antigen presentation by antigen-presenting cells among PBMCs depleted of T cells. The cocultures were initiated with PBMCs and carboxyfluorescein succinimidyl ester (CFSE)-labeled CD4^+^ T cells to assess the proliferation of CD4^+^ TCM (CD3^+^CD4^+^CD45RO^+^CD62L^+^) and CD4^+^ TEM (CD3^+^CD4^+^CD45RO^+^CD62L^−^) cells using flow cytometry. Collecting PBMCs and CFSE-labeled CD4^+^ TCM cells 7 days after the initiation of coculture revealed slight increases in the cell populations for YiP3 cells and clones A7 and B2, with average increases of 4.8%, 4.5% and 4.7%, respectively. However, upon restimulation of YiP3 cells and clones A7 and B2 with IFN-γ-stimulated PBMCs after 14 and 21 days of coculture, respectively, the proliferation of CFSE-labeled CD4^+^ TCM cells increased to 12.8% (day 14, mean) and 25.2% (day 21, mean) for YiP3 cells. By contrast, proliferation was reduced to 9.6% (day 14, mean) and 19.3% (day 21, mean) for clone A7, which was significantly lower than that of YiP3 cells (*P* < 0.05 versus YiP3). Clone B2 demonstrated a similar trend of increased proliferation, but the reduction was less pronounced than that of clone A7, with no significant difference in *P* values observed (Fig. 6c, d). Similar results were observed in CFSE-labeled CD4^+^ TEM cells. On day 21 of coculture, the proliferation increased to 22.7% (day 21, mean) for CD4^+^ TEM cells in response to YiP3 stimulation, whereas clone A7 exhibited a significantly lower proliferation of 17.1% (day 21, mean) compared with that of YiP3 cells (*P* < 0.05 versus YiP3). Clone B2 did not significantly differ from YiP3 cells (Fig. 6c, d).

These results indicate that triple-KO clone A7, with edited *HLA-A*, *HLA-B* and *HLA-DRA* genes, exhibited reduced immunogenicity when cocultured with PBMCs of different HLA types, indicating its potential for immunological compatibility.

To further investigate the immunogenicity of gene-edited iPS cells, we conducted coculture experiments using NK cells derived from donor B, whose HLA types differed from those of YiP3 cells (Fig. 6e). NK cells were isolated from donor B PBMCs, stimulated with IL-2 and then cocultured with YiP3, A7 and B2 cells. Before coculture, YiP3, A7 and B2 cells were pretreated with IFN-γ. After 6 h of coculture, the population of cells positive for CD107a, an activation marker of NK cells, was measured via flow cytometry (Fig. 6f). The results revealed that the percentages of CD107a-positive cells increased to 6.71% with unedited YiP3 cells, 1.37% with A7 cells and 3.66% with B2 cells. The NK cell activity was significantly reduced with A7 cells (*P* < 0.01 versus YiP3), although the reduction was less pronounced with B2 cells (*P* < 0.05 versus YiP3) (Fig. 6g). Because CD107a expression on NK cells occurs when cytotoxic granules are released to kill target cells, it is linked to immunogenicity. Therefore, the triple-KO clone A7, which retains HLA-C while having edited HLA-A, HLA-B and HLA-DRA genes, significantly reduced NK cell activity compared with that of YiP3 cells, indicating decreased immunogenicity.

### Assessment of HLA protein expression in differentiated cells

**Fig. 7:**
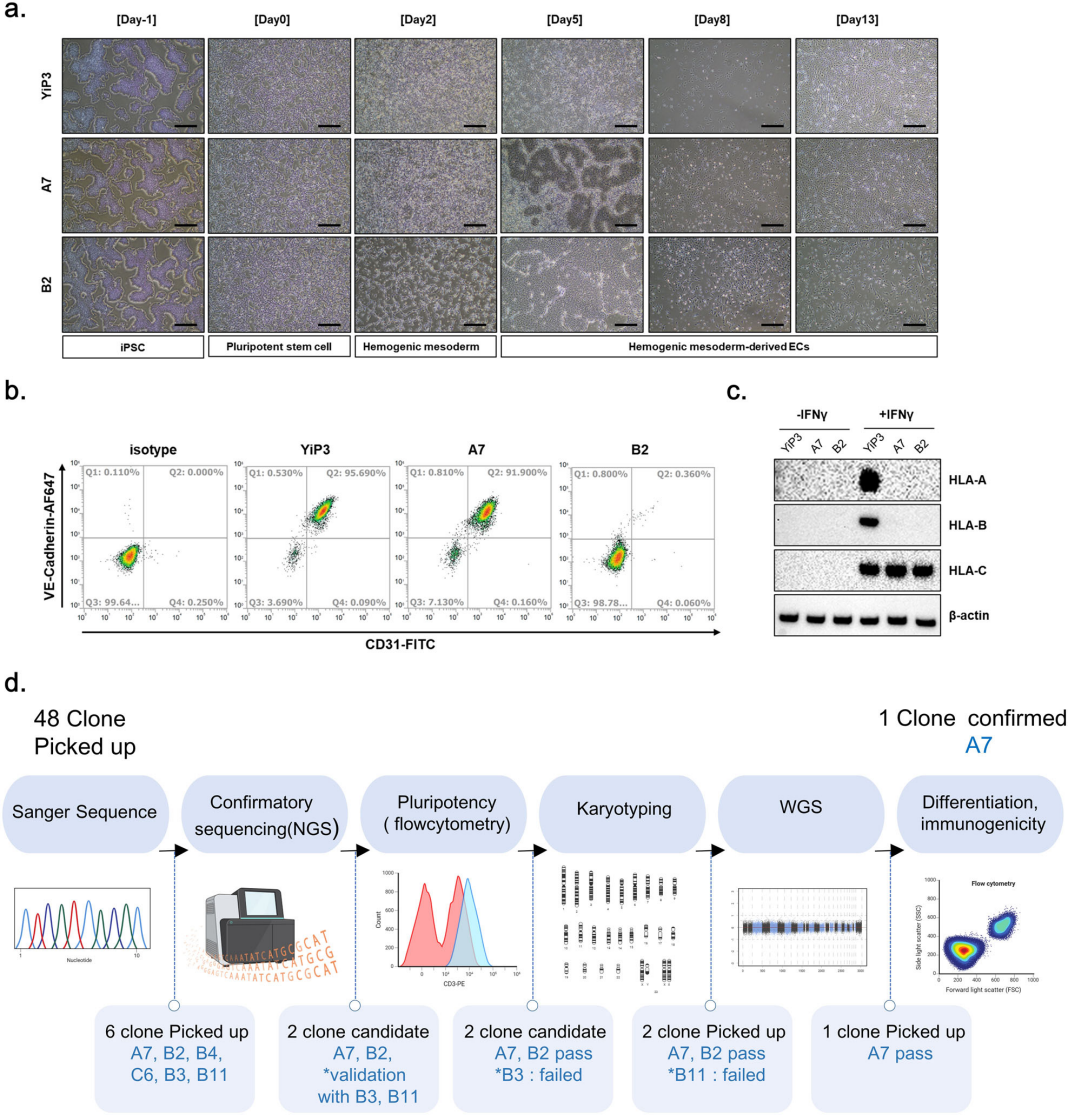
Assessment of endothelial differentiation capability and validation of HLA protein expression in triple-*HLA*-KO iPS-cell-derived ECs. **a**, Microscopy images after EC differentiation using YiP3 and clones A7 and B2. Scale bars, 500 μm. **b**, Flow cytometry data showing the cell population expressing the EC markers CD31 and VE-cadherin after EC differentiation of YiP3 and clones A7 and B2. **c**, The western blotting data showing alterations in the protein expression levels of HLA-A, HLA-B and HLA-C before and after IFN-γ stimulation after EC differentiation of YiP3 and clones A7 and B2. **d**, A schematic diagram illustrating the process of selecting triple-KO clones for *HLA-A*, *HLA-B* and *HLA-DRA* after gene correction. This illustration was created using bioRender.

We differentiated clones A7 and B2 into ECs of the mesoderm lineage and compared their differentiation capacities. Both YiP3 cells and clone A7 differentiated into ECs through the hemogenic mesoderm, which displayed a morphology similar to that of primary ECs. However, we observed a slightly different morphology for clone B2 (Fig. 7a). Flow cytometry analysis of CD31 and VE-cadherin double-positive cells, which are markers of ECs, revealed percentages of 95.69, 91.9 and 0.36% for YiP3 cells and clones A7 and B2, respectively (Fig. 7b). These results indicated that clone A7, with a differentiation rate of over 90% in ECs, exhibited normal differentiation ability. By contrast, clone B2 exhibited a diminished capacity to differentiate into ECs, as shown in Fig. 5f.

Western blot analysis revealed that before IFN-γ stimulation, HLA-A, HLA-B and HLA-C were not expressed in ECs derived from YiP3 cells and clones A7 and B2. However, after IFN-γ stimulation, HLA-A, HLA-B and HLA-C were expressed in YiP3 cells, whereas only HLA-C was expressed in clones A7 and B2, which indicated that HLA-A and B were not expressed (Fig. 7c). This confirmed that clone A7 retained its ability to differentiate into ECs, and under IFN-γ stimulation, HLA-A and HLA-B were not expressed, whereas HLA-C, which is not the target of gene editing, was expressed. We differentiated YiP3 and clone A7 cells into ECs (iECs), sensory neurons (iSNs), and hepatocytes (iHeps) and analyzed the mRNA expression levels of differentiation markers. In ECs, the mRNA expression levels of platelet EC adhesion molecule, cadherin-5 (*CDH5*), *TEK* and kinase insert domain receptor (*KDR*) were significantly increased, whereas that of *OCT4* was reduced (Supplementary Fig. 5a). Similarly, sensory neuron differentiation was associated with increased perinipherin (*PRPH*) and SRY-related HMG-box (*SOX10*) expression, with a reduction in *OCT4* (Supplementary Fig. 5b). Western blot analysis confirmed that A7 expressed the sensory neuron markers CACNA2D1, NAV1.7 and peripherin, similar to YiP3 cells (Supplementary Fig. 5c). In hepatocytes, the mRNA levels of albumin (*ALB*) and *SERPINA1* were elevated, whereas those of *OCT4* were decreased (Supplementary Fig. 5d). Western blot analysis also revealed that A7 expressed the hepatocyte markers alpha-fetoprotein (AFP), alpha-1 antitrypsin and ALB (Supplementary Fig. 5e). These findings confirmed that, similar to YiP3 iPS cells, triple-KO A7 iPS cells also retain pluripotency and can differentiate into all three lineages.

In addition, when the mRNA expression levels of *HLA-A*, *HLA-B* and *HLA-DRA* in differentiated hepatocytes were measured, the expression levels of all three markers were significantly lower in hepatocytes differentiated from A7 than in those differentiated from YiP3 cells (*HLA-A*: *P* < 0.001 versus YiP3, *HLA-B*: *P* < 0.01 versus YiP3) and *HLA-DRA*: *P* < 0.01 versus YiP3) (Supplementary Fig. 5f).

We performed a transcriptome analysis of cells differentiated into hepatocytes from YiP3 and clone A7 cells and analyzed their differentiation-related functions (Supplementary Fig. 6). *ALB*, *SERPINA1*, *AFP* and *CYP3A4*, which are known hepatocyte markers, were specifically expressed in hepatocytes differentiated from YiP3 and clone A7 cells (Supplementary Fig. 6a). Moreover, the expression levels of *CEBPA*, *KRT18* and *KRT8* were significantly greater in hepatocytes than in iPS cells. In both A7 cells and A7-derived hepatocytes, the expression levels of *HLA-A*, *HLA-B* and *HLA-DRA*, which were knocked out, were reduced (Supplementary Fig. 6b). This transcriptome analysis confirmed that hepatocytes derived from YiP3 and clone A7 cells exhibit a high degree of transcriptomic similarity (Pearson correlation coefficient, *r* = 0.98) (Supplementary Fig. 6c). Moreover, gene set enrichment analysis revealed significant enrichment of functions associated with hepatocytes and the liver, such as hepaticobiliary system development, liver regeneration and bile acid metabolic processes (Supplementary Fig. 6d), and hepatocyte- and liver-related genes were significantly enriched at the transcriptome level in hepatocyte samples derived from YiP3 and clone A7 cells (Supplementary Fig. 6e). Our results demonstrate that genome-edited iPS cell A7 cells, selected through the process of triple-KO clone selection, as shown in the scheme in Fig. 7d, have the capacity to differentiate into specific cell types.

## Discussion

In this study, we aim to generate immune-evasive iPS cells for gene editing using iPS cells with heterogeneous *HLA* gene types. Using the CRISPR–Cas9 system, we knocked out the *HLA-A*, *HLA-B* and *HLA-DRA* regions. Although previous research has focused on editing *HLA* genes using homozygous or partially homozygous iPS cells, the majority of iPS cells used in practical applications are derived from donor somatic cells with heterozygous *HLA* genes^38^. In this study, we attempted gene editing in PBMC-derived iPS cells with heterogeneous alleles in the *HLA* region, addressing the technical challenge of editing three genes (*HLA-A*, *HLA-B* and *HLA-DRA*) that can be spliced by a common gRNA. The selected triple-KO clone (clone A7) demonstrated gene KO at the RNA level and confirmed the absence of HLA-A, HLA-B and HLA-DR proteins upon IFN-γ stimulation at the protein level, which was observed by flow cytometry. Moreover, we demonstrated that clone A7 retained pluripotency after gene editing. Moreover, when CD4^+^ T cells obtained from PBMCs, which have different HLA types than YiP3 cells, were cocultured alongside IFN-γ-stimulated triple-KO clone A7, the proliferation of TCM and TEM cells was significantly lower than that of unedited YiP3 iPS cells, confirming the absence of immune evasion in clone A7. In addition, in supplementary experiments, when NK cells from a donor with a different HLA type were cocultured, NK activation, as indicated by the CD107a-positive cell population, was significantly reduced in A7 cells compared with YiP3 cells, where HLA-C was retained and HLA-A, HLA-B and HLA-DRA were knocked out.

Recent research has focused on generating universal iPS cells by editing *HLA* genes. In addition, studies have aimed to edit *HLA* genes in embryonic stem cells^42^ and completely eliminate MHC class I genes using *β2M* KO and MHC class II genes using *CIITA* KO in iPS cells^43,44^. Recent studies have focused on selecting^45^ and knocking out one *HLA* gene at a time^36^. In this study, considering the risk of NK cell or macrophage attacks that may occur in cases where all *HLA* genes are eliminated, we chose to retain minor *HLA-C* and select *HLA-A* and *HLA-B* for KO, while implementing a strategy to knock out *HLA-DRA* to eliminate HLA-DR protein expression. Although previous studies have focused on knocking out *HLA-DRB1*, we disrupted the protein structure of HLA-DR by knocking out the *HLA-DRA* gene. To design a gRNA for KO, we used the immuno polymorphism database-international immunogenetics information system (IPD-IMGT)/HLA database, which reflects the polymorphisms of *HLA* genes. The validation of gRNA design ensured the selection of common regions shared by both alleles.

To assess the KO efficiency of gRNA candidates, we initially performed electroporation followed by Sanger sequencing. One gRNA was selected for each *HLA-A*, *HLA-B* and *HLA-DRA* gene. To attempt triple-KO using the selected gRNAs, we determined the amount of RNP to be transfected and executed the procedure accordingly. Upon confirming the KO efficiency through Sanger sequencing, we obtained clone A7, which exhibited a 1-bp insertion, 28-bp deletion and 1-bp deletion in the *HLA-A*, *HLA-B* and *HLA-DRA* regions, respectively. To select the final triple-KO clone, we initially selected 48 clones and confirmed clone A7 through NGS, pluripotency assays, karyotyping and WGS^46^. During the clone selection process, clones A7 and B2 emerged as strong candidates based on NGS analysis, with B3 and B11 as secondary options. Because triple-KO iPS cells should retain pluripotency after gene editing, iPS cell quality control was conducted. Among the candidate clones, B3 was excluded because of its significantly low NANOG expression in protein analysis after gene editing. By contrast, B11 was disregarded because of abnormal karyotyping results. The candidate clones excluded from the clone selection process displayed normal morphology; however, they presented different outcomes than those of YiP3 cells in terms of quality control parameters, such as pluripotency, karyotype and NGS analysis. To further compare clones A7 and B2, they were differentiated into ECs of a mesoderm lineage. When the differentiation rates were compared, clone A7 presented a differentiation rate similar to that of pregene-edited YiP3 cells, indicating that triple-KO did not affect differentiation. However, clone B2 exhibited a significantly lower efficiency in EC differentiation, resulting in its exclusion from the final clone selection.

HLA protein expression was not detected in iPS cells or differentiated cells. In this study, we assessed HLA expression in iPS cells upon IFN-γ stimulation, which marked the first instance of this phenomenon^47^. The flow cytometry results revealed that clones A7 and B2 did not express HLA-A, HLA-B, or HLA-DR proteins upon IFN-γ stimulation. HLA-DR exhibited consistently low protein expression in iPS cells, which did not significantly increase upon IFN-γ stimulation but was reduced after gene editing. Before IFN-γ stimulation, the HLA-A and B proteins are not expressed during EC differentiation^40^. However, YiP3 cells expressed these genes before gene editing, whereas clones A7 and B2 presented no expression. HLA-C expression was retained throughout the study period. However, HLA-DR and DRA expression was not confirmed upon IFN-γ stimulation in differentiated ECs. Although HLA-DR protein expression has been reported in ECs^48,49^, it was not detected in differentiated ECs. Because of its abundance in immune cells^50^, further assessment is needed. Through additional assays, we differentiated A7 cells into hepatocytes and confirmed through transcriptome analysis that the mRNA expression levels of HLA-A, HLA-B and HLA-DRA in A7-derived hepatocytes were consistently downregulated.

To use universal iPS cells as cell therapeutics, it is essential to conduct immunogenicity testing. In this study, we performed in vitro immunogenicity tests instead of mouse models because of the challenges in studying memory T cells owing to interspecies differences^51,52^. We used PBMCs from donors with different HLA types from the HLA type of YiP3 cells before gene editing. CD4^+^ T cells were labeled with CFSE to assess their proliferation in response to stimulation with YiP3 cells and clones A7 and B2, which served as antigenic stimuli. T cell activation was initiated using IFN-γ-stimulated iPS cells 2 days prior, and the proliferation of TCMs and TEMs was examined. We observed reduced proliferation levels in clone A7 compared with those in YiP3 cells, indicating that clone A7 failed to induce T cell proliferation because of the absence of *HLA-A*, *HLA-B* and *HLA-DRA* expression.

iPS cells have the potential to differentiate into various cell types, including ECs, neurons, cardiac cells and immune cells^53^. Because of their ability to proliferate indefinitely and differentiate into all three-germ layers, iPS cells exhibit immense potential for use in cell therapies. Clinical trials involving the transplantation of allogeneic iPS cells have focused primarily on immune-privileged sites, such as the eye and cartilage^54–56^. However, for transplantation into tissues associated with immune responses, such as the cardiovascular system, immune cells and kidneys, it is crucial to use hypoimmunogenic iPS cells with immune-invasive properties as source cells^57^.

This study demonstrates the selection of clones that selectively knock out the *HLA-A*, *HLA-B* and *HLA-DRA* genes through various quality control measures, indicating their suitability for therapeutic applications. These *HLA*-KO iPS cell clones hold promise for cell therapy and the development of iPS-cell-derived organoid therapies. While currently used for research purposes, advancing these iPS cells to clinical-grade status involves production within GMP facilities and adherence to regulatory science standards^58–60^. With such advancements, it is conceivable that clinical-grade universal iPS cells may be developed for research and development purposes.

We generated hypoimmunogenic iPS cell clone A7 by knocking out the *HLA-A*, *HLA-B* and *HLA-DRA* genes in PBMC-derived iPS cells with heterozygous *HLA* gene types. We confirmed that this clone retains the pluripotency of iPS cells and verified its genetic stability using various sequencing methods. Our results establish a strategy and quality standards for selectively using triple-KO iPS cells lacking *HLA-A*, *HLA-B* and *HLA-DRA* as universally accepted iPS cells devoid of immunogenicity. Our findings offer important criteria and methodologies for the future development of clinical-grade universal iPS cells.

## Supplementary Information

Supplementary Information Supplementary Figs. 1–6, Tables 1–6 and methods.
