# Supplementary material for: Generation of hypoimmunogenic universal iPS cells through HLA-type gene knockout
Source: Exp Mol Med. 2025 Mar 14;57(3):686–99. doi: 10.1038/s12276-025-01422-3 (PMC11958689; doi:10.1038/s12276-025-01422-3)
Supplement: Supplementary file 1 — Supplementary Information Supplementary Figs. 1–6, Tables 1–6 and methods. [file 12276_2025_1422_MOESM1_ESM.docx]

This word file include:

Supplementary Materials and Methods Information

Supplementary Tables (Table 1-6)

Supplementary Figures and Figure Legends (Supplementary Figure and Figure Legend 1-6)

**Supplementary Materials and Methods**

**iPSC culture**

The gene-edited cell lines were thawed and passaged following the manufacturer’s protocol. Frozen induced pluripotent stem cells (iPSCs) were thawed rapidly in a 37 ⁰C water bath. Subsequently, cryovials were gently transferred to a conical tube containing a pre-warmed mTeSR-Plus culture medium (100-0276; STEMCELL). Following centrifugation for 3 min at 200 x g at room temperature (RT), the appropriate number of cells was plated onto a Matrigel (354277; Corning)-coated 6-well culture plate containing 10 µM Y-27632 (1293823; Biogems). Once the colonies were stabilized, fresh culture medium was replaced daily. To passage iPSCs, the cells were dissociated using 1X TrypLE Express Enzyme (12604013; Thermo Fisher). The total cell number was counted, and an appropriate number of cells was plated on a Matrigel-coated T75 flask with 10 µM Rho-associated kinase inhibitor (ROCKi). Subsequently, the medium was replaced daily with fresh mTeSR-Plus.

After the cells were stabilized, the coating material and culture medium were replaced to culture the cells under identical conditions similar to those of the control iPSCs. When passaging the gene-edited iPSCs, the cells were plated in vitronectin (A14700; Invitrogen)-coated T75 culture flasks containing 10 µM Rho-associated protein kinase inhibitor (ROCKi). The following day of ROCKi withdrawal, the medium was replaced with a 1:1 ratio of mTeSR-Plus and Essential 8 medium (Gibco, 1517001), followed by the complete replacement of the medium with fresh Essential 8 medium. Subsequently, the cells were cultured using the aforementioned procedure.

To culture the cells on hypoxic condition, the appropriate number of cells was plated into a VTN-N-coated T75 culture flask with 10uM ROCK inhibitor. Then, the cells were incubated in 37°C incubator under an atmosphere of <5% O_2_/5% CO_2_. The medium was daily exchanged with fresh Essential 8 medium but without the ROCK inhibitor. The cells were continuously cultured under low oxygen condition during their expansion.

**Endothelial cell differentiation**

Endothelial cells were generated as described previously. When pluripotent stem cells (PSCs) reached over 70 % confluency, cells were detached using 1X TrypLE Express Enzyme (12604039; Gibco) and resuspended by pipetting. After aspirating liquid from a variant of vitronectin (VTN)-N-coated T25 culture flask (A14700; Invitrogen), cells were plated at a density of ~1.6 x 10^5^ cells/cm^2^ containing 10 µM of Y-27632 (1293823; Biogems). On day 0, cells were treated with RPMI-1640 (11875093; Gibco), 100 ng/mL Activin A (338-AC; R&D Systems), and 1X B-27 without insulin (A1895601; Gibco) and incubated for 17 h. On day 1, the medium was replaced with RPMI-1640, 5 ng/mL BMP4 (120-05ET; Peprotech), 1 µM CHIR-99021 (2520691; Biogems), and 1X B-27 without insulin. On days 2–4, the cells were cultured in StemPro-34 SFM (10639011; Gibco), supplemented with 400 µM 2-mercaptoethanol (MTG) (M6145; Sigma), 2 mM L-glutamine (25030081; Gibco), 50 µg/mL ascorbic acid (A8960; Sigma), 10 ng/mL bone morphogenetic protein 4 (BMP-4), 5 ng/mL basic fibroblast growth factor (bFGF) (100-18B; Peprotech), and 300 ng/mL vascular endothelial growth factor (VEGF) (100-20; Peprotech). On day 5, the cells were split using 1X TrypLE Express Enzyme and replated onto the VTN-N-coated T25 culture flasks at a density of 9.0 x 10^3^ cells/cm^2^, using endothelial growth medium (EGM) (CC-3124; LONZA), supplemented with 20 ng/mL VEGF, 20 ng/mL bFGF, and 1 µM CHIR-99021.

**Hepatocyte differentiation**

To differentiate definitive endoderm from iPSCs, the cells were plated on (VTN)-N-coated 6 well culture plate at a density of 2.0x10^6^ cells with 10μM of Y-27632. On day 1, the cells were treated with RPMI-1640 (Gibco, 11875093), 2mM L-Glutamine (Gibco, 25030081), 100ng/mL Activin A (R&D, 11348-AC), 20ng/mL hWNT3A (R&D, 5036-WN). On day 2, media were exchanged with 2mM L-Glutamine supplemented fresh RPMI-1640 containing 100ng/mL Activin A, 8ng/mL bFGF (Peprotech, 100-18B), 0.2% defined FBS (Gibco, 10099141). On Day 3, media were also exchanged with 2mM L-Glutamine supplemented fresh RPMI-1640 containing 100ng/mL Activin A, 8ng/mL bFGF, 2% defined FBS. On Day 4-6, the plate was washed with RPMI-1640 media, and then media was replaced with RPMI-1640 supplemented 1X B-27 (Gibco, 17504001), 10ng/mL bFGF, 20ng/mL BMP-4 (Peprotech, 120-05ET). On Day 8, the medium was replaced every other day with hepatocyte basal medium (LONZA, CC-3198) containing 50ng/mL HGF (Peprotech, 100-39H) and 30ng/mL Oncostatin M (Peprotech, 300-10) until Day 30.

**Sensory neuron differentiation**

iPSCs were seeded at a density of 6 x 10⁴ cells/cm², and the culture medium was replaced with neural crest cell differentiation medium starting the following day. Neural crest cell differentiation media 1 and 2 were mixed in specific ratios based on the differentiation day and used for medium exchanges. Medium 1 was used at 100% until Day 3, at 75% on Days 4-5, at 50% on Days 6-7, at 25% on Days 8-9, and at 0% from Day 10 onwards, with the remaining volume made up by Medium 2. SB431542 (1614, Tocris) and Dorsomorphin (3093, Tocris) were added from Day 0 to Day 6, while DAPT (D5942, Sigma), SU5402 (SML0443, Sigma), and CHIR99021 (SML1046, Sigma) were added from Day 3 to Day 11 during medium changes.

Neural crest cell differentiation medium 1 was prepared by combining 500 ml of Knockout DMEM CTS (10829018, Gibco), 130 ml of Knockout SRXENOFREE CTS (12618013, Gibco), 1x MEM Non-essential Amino Acid Solution (11140050, Gibco), 1x Glutamax,(A1286001, Gibco) and 0.01 mM β-Mercapto-ethanol (21985023, Gibco).

Neural crest cell differentiation medium 2 was prepared using 500 ml of Neurobasal Medium minus phenol red (12348017, Gibco), 1x N-2 Supplement (17502001, Gibco), 1x B-27 Supplement XenoFree minus Vitamin A (**12587001**, Gibco), and GlutaMAX-1 (**35050061**, Gibco) and 0.01 mM β-Mercapto-ethanol (21985023, Gibco).

To induce peripheral neurons from differentiated neural crest cells, the culture medium was replaced with Neural Crest Cell Differentiation Medium 2 supplemented with 25 ng/ml of BDNF (AF-450-02, Peprotech), GDNF (AF-450-10, Peprotech), NT-3 (AF-450-03, Peprotech), and b-NGF (AF-450-01, Peprotech). The medium was refreshed every 4 to 5 days until Day 35.

**Alkaline phosphatase (AP) staining**

For alkaline phosphatase staining, iPSCs were plated at low density onto VTN-N-coated 6-well culture plates. After the colonies reached the desired size over 5 days, the wells were rinsed with 1 mL of 0.05 % phosphate-buffered saline with Tween 20 (PBST) and fixed in 1 mL of 4 % paraformaldehyde for 10 min at RT. The cells were washed with 1 mL of 0.05 % PBST, and subsequently 1 mL of staining solution was added, which comprised Fast Red Violet, Naphthol AS-BI phosphate solution, and triple-distilled water (TDW) (SCR004; Sigma). The cells were incubated for 30 min at RT in the dark. The cells were rinsed once with 0.05 % PBST and twice with 1x Dulbecco's phosphate-buffered saline (DPBS). Images were visualized using a Nikon ECLIPSE Ts2 microscope.

**Fluorescence-activated cell sorting (FACS) analysis**

For flow cytometry analysis, the cells were detached using 1X TrypLE Express Enzyme and fixed with Permeabilization and Fixation solution (554722; BD Biosciences) for 20 min at 4 ⁰C. Following two rinses with 1X Perm/Wash solution (554723; BD Biosciences), we used the following antibodies: allophycocyanin (APC)-conjugated HLA-A (568024; BD Biosciences), BV510-conjugated HLA-B (752615; BD Biosciences), phycoerythrin (PE)-conjugated HLA-C (566372; BD), fluorescein isothiocyanate (FITC)-conjugated HLA-DR (11-9956-42; Invitrogen), allophycocyanin (APC)-conjugated human CD34 (555824, BD Biosciences), fluorescein isothiocyanate (FITC)-conjugated TRA-1-60 (560380, BD Pharmingen), phycoerythrin (PE)-conjugated NANOG (560483, BD Pharmingen), PerCP-Cy5.5-conjugated SSEA-4 (561565, BD Biosciences), phycoerythrin (PE)-conjugated OCT3/4 (IC1759P-100, R&D), Alexa Fluor 647-conjugated VE-Cadherin (ab272346, Abcam), fluorescein isothiocyanate (FITC)-conjugated CD31 (555445, BD Biosciences), with respective isotype control antibodies: APC-conjugated mouse IgG isotype control (554681; BD Biosciences), BV510-conjugated rat IgG isotype control (563039; BD Biosciences), phycoerythrin (PE)-conjugated mouse IgG isotype control (555058; BD Biosciences), fluorescein isothiocyanate (FITC)-conjugated mouse IgG isotype control (11-4732-42, Invitrogen) , allophycocyanin (APC)-conjugated mouse IgG1 isotype control (555751, BD Biosciences), fluorescein isothiocyanate (FITC)-conjugated mouse IgM isotype control (555583, BD Biosciences), phycoerythrin (PE)-conjugated mouse IgG1 isotype control (555749, BD Biosciences), PerCP-CY5.5-conjugated mouse IgG1 Isotype control (550795, BD Biosciences), phycoerythrin (PE)-conjugated rat IgG2b isotype control (553989, BD Biosciences),, Alexa Fluor 647-conjugated mouse IgG1 isotype control (557732, BD Biosciences), fluorescein isothiocyanate (FITC)-conjugated mouse IgG1 isotype control (555748, BD Biosciences). These were incubated for 40 min at 4 ⁰C. The cells were rinsed twice with 1X Perm/Wash solution and subsequently analyzed using the Attune NxT instrument (Invitrogen).

**Three germ lineage differentiation assay**

The trilineage germ cell layers were differentiated using the STEMdiff Trilineage differentiation kit (05230; STEMCELL Technology), following the manufacturer’s protocol. The cells were plated into a 24-well culture plate at a density of 2.0 x 10^5^ cells/well (ectoderm and endoderm) or 1.0 x 10^5^ cells/well (mesoderm) containing 10 µM Y-27632 and 1 µg/mL iMatrix-511MG (MX892012; Nippi). To differentiate the ectoderm lineage, the medium was replaced daily for a week, whereas for mesoderm or endoderm lineage differentiation, the medium was replaced daily for only 5 days. For immunostaining, the cells were fixed with 4 % paraformaldehyde and stained following the methods described below.

**Immunofluorescence (IF) assay**

The cells were washed twice with 1X DPBS and fixed with 4 % paraformaldehyde for 30 min. After washing the cells with 1X DPBS twice, 50 mM NH_4_Cl was added for 10 min to quench autofluorescence. To permeabilize the cells, 0.1 % PBST was added for 30 min. The cells were rinsed twice with 1X DPBS and soaked in 1X DPBS-based 2 % bovine serum albumin (BSA) for 30 min at RT. Consequently, primary antibodies, such as OTX2 (AF1979, 1:100; R&D), BRACHYURY (AF2085, 1:100; R&D), and SOX17 (AF1924, 1:100; R&D) were diluted with BSA and incubated overnight at 4 ⁰C. After washing twice with BSA, the cells were incubated with Alexa Fluor 488 (A10680, 1:200; Invitrogen) or Alexa Fluor 594 (A11037, 1:200; Invitrogen)-conjugated secondary antibodies in the dark for 30 min. For counterstaining, 0.5 µg/mL of 4′,6-diamidino-2-phenylindole (DAPI) was incubated for 10 min at RT. The cells were mounted with an antifade mounting medium (H-1700; VectorLabs) and visualized using a Cytation 5 (BioTek) fluorescence imaging system.

**Western blotting**

For cell lysis, 1 mL per 5.0 x 10^6^ cells of radio-immunoprecipitation assay buffer (RIPA) lysis buffer (89901; Invitrogen) containing 1X proteinase inhibitor cocktail (78430; Invitrogen) was added to the cells on ice for 10 min. Following centrifugation (13,200 x g, 20 min), the lysates were collected in fresh tubes. Protein concentration was quantified using a bicinchoninic acid assay (BCA) kit (23227; Invitrogen). Subsequently, we mixed 4X lithium dodecyl sulfate (LDS) sample buffer (B0007; Invitrogen) and 10X reducing agent (B0009; Invitrogen) with the cell lysates based on the sample concentration and denatured them by boiling for 5 min. Each sample was separated through electrophoresis on 4–12 % Bis-Tris polyacrylamide gel electrophoresis (PAGE) gels (NW04125BOX; Invitrogen). Subsequently, the proteins were transferred to a polyvinylidene fluoride (PVDF) membrane using iBlot and blocked for1 h with 5 % skim milk to prevent non-specific binding. Primary antibodies, such as HLA-A (ab52922, 1:5 000, Abcam), HLA-B (ab193415, 1:1 000; Abcam), HLA-C (ab126722, 1:1 000; Abcam), HLA-DR (ab92511, 1:1 000, Abcam), HLA-DRA (PTG-17221-1-AP, 1:1000; Proteintech), AFP (A8452, 1:500, Sigma Aldrich), A1AT (GTX77515, 1:1,000, GeneTex), ALB (sc-271605, SantaCruz), PERIPHERIN (AB1530, 1:1,000, MilliporeSigma), CACNA2D1 (MA3-921, 1:1,000, Invitrogen), NAV1.7 (MABN41, 1:1,000, MilliporeSigma) and β-actin (sc-47778 HRP, 1:1 000; SantaCruz) were diluted in 5 % skim milk, and the membranes were incubated at 4 ⁰C overnight. The membranes were washed thrice in 1X PBST buffer on an orbital shaker, followed by a 1-hour incubation with the secondary antibodies diluted at 1:10 000. Subsequently, the membranes were washed thrice, soaked in an enhanced chemiluminescence (ECL) reagent (K-12045; Advansta), and detected using an iBright 1500 instrument (Invitrogen).

***In vitro* immunogenicity assay**

*In vitro* immunogenicity assay was performed to assess CD4+ T cell response. Human PBMCs were incubated in a culture dish (100 mm) in AIM-V medium (12055-083; Gibco) at 37 ⁰C in a 5 % CO_2_ incubator after thawing, and an appropriate concentration of DNase was added. CD4+ T cells were sorted using the CD4+ T cell Isolation Kit (130-096-533; Miltenyi). The cells were incubated with 0.5 µM carboxyfluorescein succinimidyl ester (CFSE) (C34554; Invitrogen) at 37 ºC for 10 min in the dark and washed once with 1X DPBS. CD3-/CD4- cells in CD4- PBMCs were obtained using the CD3 Microbeads (130-050-101; Miltenyi). Subsequently, Mitomycin C (10 µg/mL) was added for 20 min and the cells were washed with AIM-V medium. For co-culturing, 3.0 x 10^5^ CD3-/CD4- PBMC and 4 x 10^5^ carboxyfluorescein succinimidyl ester (CFSE)-probed CD4+ T cells were combined into a 24-well culture plate. To induce a direct homologous antigen stimulation, 3 x 10^3^ iPSCs were added to each well. Human recombinant interleukin (IL)-2 (1 ng/mL) was added, and the cells were incubated for a week at 37 ⁰C. To confirm the immunogenicity of the iPSCs, the proliferation of carboxyfluorescein succinimidyl ester (CFSE)-labeled CD4+ T cells was assessed through flow cytometry. The remaining cells were co-cultured for seven days with additional stimulation by adding 3.0 x 10^5^ CD3-/CD4- PBMCs and 3.0 x 10^3^ iPSCs. This process was assessed at weekly intervals for a total of 3 weeks to assess long-term immunogenicity.

**NK cell isolation**

PBMCs were isolated by density gradient centrifugation (Ficoll-paque^TM^ PLUS, 17144003; Cytiva). PBMCs were cultured in RPMI-1640 supplemented with glutamine (11875093; Gibco) plus 10% FBS (16141-079; Gibco) and 1% pen/strep (15140-122; Gibco) for 24 hours. NK cells were isolated from PBMCs by immunomagnetic negative selection using the EasySep^TM^ direct human NK cell isolation kit (19665; STEMCELL), according to the manufacturer’s instructions. Isolated NK cells were cultured in PBMCs culture medium in addition to 1X MEM Non-essential amino acids (11140050, Gibco) before performing the assays.

**CD107a assay**

NK cells were stimulated with IL-2 (200-02-1MG; Peprotech) 1µg/mL overnight before and during assays. Stimulated NK cells were co-cultured with YiP3, A7 and B2 cells, which were pre-treated with IFN-γ for three days at an E/T ratio of 1:1 (effector: 1.0 x 10^4^ cells, target: 1.0 x 10^4^ cells) in a 24-well culture plate for 6 hours in a final volume of 500 µL RPMI-1640 supplemented with glutamine (11875093; Gibco) plus 10% FBS (16141-079; Gibco), 1X MEM NEAA (11140050, Gibco), 1% pen/strep (15140-122; Gibco) and 20 µL of anti-CD107a antibody (555800; BD Bioscience). Finally, the percentage of CD107a-positive NK cells was measured by FACS analysis.

**Assessment of genetic stability**

**Copy number variation (CNV) analysis**

The genomic DNAs of YiP3 iPSCs (wild-type) and *HLA*-triple KO clones A7 and B2 were genotyped using the CytoScan-HD array (Thermo Fisher Scientific, Inc.), following the manufacturer’s protocol. Initially, 250 ng of genomic DNA was digested using NspI and amplified using Titanium Taq DNA polymerase (Takara Bio, Inc.). Subsequently, it was fragmented using a fragmentation reagent and labeled with biotin end-labeled nucleotides. Subsequently, the labeled DNA was hybridized to the microarray for 16–18 h. Following hybridization, the array was washed using the GeneChip Fluidics Station 450 (Thermo Fisher Scientific, Inc.), stained with GeneChip stain reagents (Thermo Fisher Scientific, Inc.), and scanned using the GeneChip System 3000 (Thermo Fisher Scientific, Inc.).

The data were analyzed using the Chromosome Analysis Suite (ChAS) (version 4.4.0.63; Thermo Fisher Scientific, Inc.). Copy number variation (CNV) was determined by assessing the log2 ratios of the sample signal against the signal from a genotyped internal reference and considering the allele difference (AD) and B-allele frequency (BAF).

**Whole-genome sequencing and bioinformatic analysis**

Sequencing libraries of YiP3, A7, and B2 were prepared from the input DNA (1 µg) using a TruSeq DNA sample prep kit following the manufacturer’s protocol (Illumina, Inc.). The sheared DNA fragments underwent end repair, A-tailing, adaptor ligation, and amplification, followed by clean-up. Subsequently, these libraries were subjected to paired-end sequencing using a 150-bp read length on the Illumina NovaSeq 6000 platform (Illumina, Inc.).

Clean reads from each sample, exhibiting a quality of > Q30 (%), were aligned with the human reference genome (GRCh38) using BWA (version 0.7.17). PCR duplicates were eliminated using MarkDuplicates of the Picard tool (version 2.25.8). To optimize read mapping quality, base quality score recalibration was conducted using the BaseRecalibrator Tool in GATK (version 4.1.8.1). Somatic mutations in *HLA*-triple KO clones A7 and B2 were detected using MuTect2 (version 4.1.8.1) with wild-type YiP3 serving as the reference. To balance specificity, standard Mutect2 somatic variant filters were applied to eliminate background germline variations and sequencing artifacts using the Genome Aggregation Database (gnomAD) germline resource (https://gnomad.broadinstitute.org/). Additional filters included checking for cross-sample contamination (CalculateContamination, GATK) and filtering for possible read-orientation sequencing artifacts (CollectF1R2Counts and LearnReadOrientationModel, GATK). High-confidence somatic mutations were characterized as a subset of somatic variant calls fulfilling the following criteria: (1) biallelic single nucleotide variants (SNVs) and insertion-deletion (InDels), (2) mapped read depth of ≥ 15, (3) a minimum alternate read depth of ≥ 5, and (4) exclusion of germline-like heterozygous single nucleotide variants (SNVs). The identified somatic variants were annotated using the Ensembl variant effect predictor (VEP) (version 108) and cross-referenced against the ClinVar and the Catalogue Of Somatic Mutations In Cancer (COSMIC) (version 98) databases. CNV was also detected in the whole-genome sequence data using CNVKit (version 0.9.10). We used Cas-OFFinder (http://www.rgenome.net/cas-offinder/) to predict the potential of CRISPR/Cas9 off-targets. The positions of predicted off-targets and structural variants that may be induced by on-targets and/or off-targets were visualized using a Circos plot (https://circos.ca/).

**RNA-seq and bioinformatic analysis**

cDNA libraries were prepared from total RNA (1 μg) of each sample using the TruSeq Stranded mRNA sample prep kit (Illumina, Inc.), following the manufacturer’s protocols. Following qPCR assessment, libraries were subjected to paired-end sequencing using a 150-bp read length on an Illumina NovaSeq 6000 platform (Illumina, Inc.).

Clean reads with quality scores > Q30 were aligned with the human reference genome (GRCh38) using STAR (version 2.7.1a) with the default parameters. Gene expression was quantified using Cufflinks (version 2.2.1), and fragments per kilobase of transcript per million mapped reads (FPKM) were calculated as the expression values. The differentially expressed genes (DEGs) were identified by Cuffdiff with the cutoff set at *p* < 0.01 and > 2-fold change. Gene ontology (GO) enrichment analysis for DEGs was conducted using the DAVID Gene Functional Classification Tool (http://david.abcc.ncifcrf.gov; version 2021) with the cutoff of an EASE score < 0.05.

**Supplementary Tables**

**Table 1. The primer list of pluripotency markers, three germ layer differentiation markers, and human leukocyte antigen (*HLA*)**

| **Genes** | **Direction** | **Sequence** |
| --- | --- | --- |
| *NANOG* | Forward | GATTTGTGGGCCTGAAGAAA |
|  | Reverse | CAGATCCATGGAGGAAGGAA |
| *OCT4* | Forward | ACCCCTGGTGCCGTGAA |
|  | Reverse | GGCTGAATACCTTCCCAAATA |
| *SOX2* | Forward | ATGGGTTCGGTGGTCAAGTC |
|  | Reverse | CTGATCATGTCCCGGAGGTC |
| *LIN28* | Forward | GTTCGGCTTCCTGTCCAT |
|  | Reverse | CTGCCTCACCCTCCTTCA |
| *KLF4* | Forward | TTCCCATCTCAAGGCACAC |
|  | Reverse | GGTCGCATTTTTGGCACT |
| *PAX6* | Forward | GTGTCCAACGGATGTGTGAG |
|  | Reverse | CTAGCCAGGTTGCGAAGAAC |
| *BRACHYURY* | Forward | AATTGGTCCAGCCTTGGAAT |
|  | Reverse | CGTTGCTCACAGACCACA |
| *SOX17* | Forward | CGCACGGAATTTGAACAGTA |
|  | Reverse | GGATCAGGGACCTGTCACAC |
| *HLA-A* | Forward | AGATACACCTGCCATGTGCAGC |
|  | Reverse | GATCACAGCTCCAAGGAGAACC |
| *HLA-B* | Forward | CTGCTGTGATGTGTAGGAGGAAG |
|  | Reverse | GCTGTGAGAGACACATCAGAGC |
| *HLA-C* | Forward | GGAGACACAGAAGTACAAGCGC |
|  | Reverse | ACATCCTCTGGAGGGTGTGAGA |
| *HLA-DRA* | Forward | AGCTGTGGACAAAGCCAACCTG |
|  | Reverse | CTCTCAGTTCCACAGGGCTGTT |
| *PRPH* | Forward | CAAGCAGGAGATGAACGAGTCC |
|  | Reverse | TCCAGCTCTCTCAACTGCCTGA |
| *SOX10* | Forward | ATGAACGCCTTCATGGTGTGGG |
|  | Reverse | CGCTTGTCACTTTCGTTCAGCAG |
| *PECAM1* | Forward | AAGTGGAGTCCAGCCGCATATC |
|  | Reverse | ATGGAGCAGGACAGGTTCAGTC |
| *CDH5* | Forward | GAAGCCTCTGATTGGCACAGTG |
|  | Reverse | TTTTGTGACTCGGAAGAACTGGC |
| *TEK* | Forward | GGTCAAGCAACCCAGCCTTTTC |
|  | Reverse | CAGGTCATTCCAGCAGAGCCAA |
| *KDR* | Forward | GGAACCTCACTATCCGCAGAGT |
|  | Reverse | CCAAGTTCGTCTTTTCCTGGGC |
| *ALB* | Forward | GATGAGATGCCTGCTGACTTGC |
|  | Reverse | CACGACAGAGTAATCAGGATGCC |
| *SERPINA1* | Forward | TCTGAAGAGCGTCCTGGGTCAA |
|  | Reverse | GATGGTCAGCACAGCCTTATGC |
| *GAPDH* | Forward | ACCCACTCCTCCACCTTTGA |
|  | Reverse | CTGTTGCTGTAGCCAAATTCGT |

**Table 2. Primer sequence for Sanger sequencing (human leukocyte antigen [*HLA*]-*A*)**

|  | **Primer name** | **Sequence** | **PCR product** |
| --- | --- | --- | --- |
| PCR Primer | G0002-HLA-A-947-F | GGAGGGAAACCGCCTCTGC | 947bp |
| PCR Primer | G0002-HLA-A-947- R | GGAGATCTACAGGCGATCAGGG |  |
| Sequencing primer | G0002-HLA-A-Seq-F | TCTTCACATCCGTGTCCCG |  |
| Sequencing Primer | G0002-Seq-R | ACTTGCGCTTGGTGATCTGA |  |

**Table 3. Primer sequence for Sanger sequencing (human leukocyte antigen [*HLA*]-*B*)**

|  | **Primer name** | **Sequence** | **PCR product** | |
| --- | --- | --- | --- | --- |
| Exon 2 | | | | |
| PCR Primer | G0002-HLA-B-exon2-F | ACTTGTGTCGGGTCCTTCTTC | 714bp | |
| PCR Primer | G0002-HLA-B-exon2-R | CTCGGACCCGGAGACTCG |  |  |
| Sequencing primer | G0002-HLA-B-Seq-F | TCAGAGTCTCCTCAGACGCC |  | |
| Exon 3 | | | | |
| PCR Primer | G0002-HLA-B-exon3-F | GGCTACTACAACCAGAGCGAG | 822bp | |
| PCR Primer | G0002-HLA-B-exon3-R | GAAAAGTCACGGTTCCCAAGG |  |  |
| Sequencing primer | G0002-HLA-B-exon3- Seq-F | GTCGCCCCGAGTCTCCG |  | |
| Sequencing primer | G0002-HLA-B-exon3- Seq-R | GAAAAGTCACGGTTCCCAAGG |  | |
|  |  |  |  |  |

**Table 4. Primer sequence for Sanger sequencing (human leukocyte antigen [*HLA*]-*DRA*)**

|  | **Primer name** | **Sequence** | **product size** |
| --- | --- | --- | --- |
| Exon 2 | | | |
| PCR Primer | G0002-HLA-DRA-Exon2-F | GCCCGGGTAAAGAAAGTGAGAG | 754bp |
| PCR Primer | G0002-HLA-DRA-Exon2-R | GAATTTGGGGCTTGTTAATGGC |  |
| Sequencing primer | G0002-HLA-DRA-exon2- Seq-F | ACTCTGGGTTCTTTAGCCCTC |  |
| Sequencing primer | G0002-HLA-DRA-exon2- Seq-R | GTTGGCCAATGCACCTTGAG |  |
| Exon 3 | | | |
| PCR Primer | G0002-HLA-DRA-exon 3-F | GGGGGTGCTGTCAGAGATTG | 748bp |
| PCR Primer | G0002-HLA-DRA-exon3-R | GGGAAATAAGGCAGAGTACATGGT |  |
| Sequencing primer | G0002-HLA-DRA-exon3- Seq-F | CGTTTGTACCACAATTGAGCATGG |  |
| Sequencing primer | G0002-HLA-DRA-exon3- Seq-R | CACCGAGTTTCACACAAGCATC |  |

**Table 5. Variants associated with genome editing detected from whole-genome sequencing (WGS) data of clones A7 and B2**

| Clone | Structural  variant | Detail of variant type | Size of variant  (bp) | Position (bp) on chromosome | Related gene |
| --- | --- | --- | --- | --- | --- |
| A7 | Deletion | c.170_197del  (p.Phe57SerfsTer11) | 28 bp | Chr6:29,942,853-  29,942,880 | *HLA-A* |
| B2 | Deletion | c.165_198del (p.Gln56ArgfsTer10) | 34 bp | Chr6:29,942,847-  29,942,880 | *HLA-A* |
| B2 | Deletion | CNV loss | 636,211 bp | Chr2:137,427,785-  138,063,995 | *THSD7B ~*  *HNMT* |
| B2 | Deletion | CNV loss | 1,085,752 bp | Chr6:31,356,818-  32,442,567 | *HLA-B ~*  *HLA-DRA* |

*fs, frame-shift; Ter, termination

**Table 6. Detection of somatic variants from whole-genome sequencing (WGS) data**

| Variants | No. of SNV | | | No. of InDel | | |
| --- | --- | --- | --- | --- | --- | --- |
|  | YiP3:  Donor vs WT | WT vs A7 | WT vs B2 | YiP3:  Donor vs WT | WT vs A7 | WT vs B2 |
| Variant type (total no. of variant) | 90 | 18 | 21 | 89 | 22 | 26 |
| Introns | 78 | 17 | 18 | 78 | 17 | 20 |
| 5'-/3'-UTRs | 11 | 1 | 3 | 11 | 3 | 4 |
| Coding exons | 1 | 0 | 0 | 0 | 2 | 2 |
| Synonymous variants | 0 | 0 | 0 | 0 | 0 | 0 |
| Variant related to functional effects | 1 | 0 | 0 | 0 | 2 | 2 |
| Missense (+ start lost, stop lost) | 1 | 0 | 0 | 0 | 0 | 0 |
| Frame-shift | 0 | 0 | 0 | 0 | 2 | 2 |
| In-frame | 0 | 0 | 0 | 0 | 0 | 0 |
| Nonsense | 0 | 0 | 0 | 0 | 0 | 0 |
| Splice site | 0 | 0 | 0 | 0 | 0 | 0 |
| Variant annotation (total no. of variant) | 0 | 0 | 0 | 0 | 0 | 0 |
| PolyPhen2/SIFT | 0 | 0 | 0 | 0 | 0 | 0 |
| ClinVar mutation | 0 | 0 | 0 | 0 | 0 | 0 |
| Mutation in COSMIC Tier 1 gene | 0 | 0 | 0 | 0 | 0 | 0 |

*WT, Passage 15 of YiP3

**Supplementary Figures and Figure Legends**

**Supplementary Fig. 1.**

**
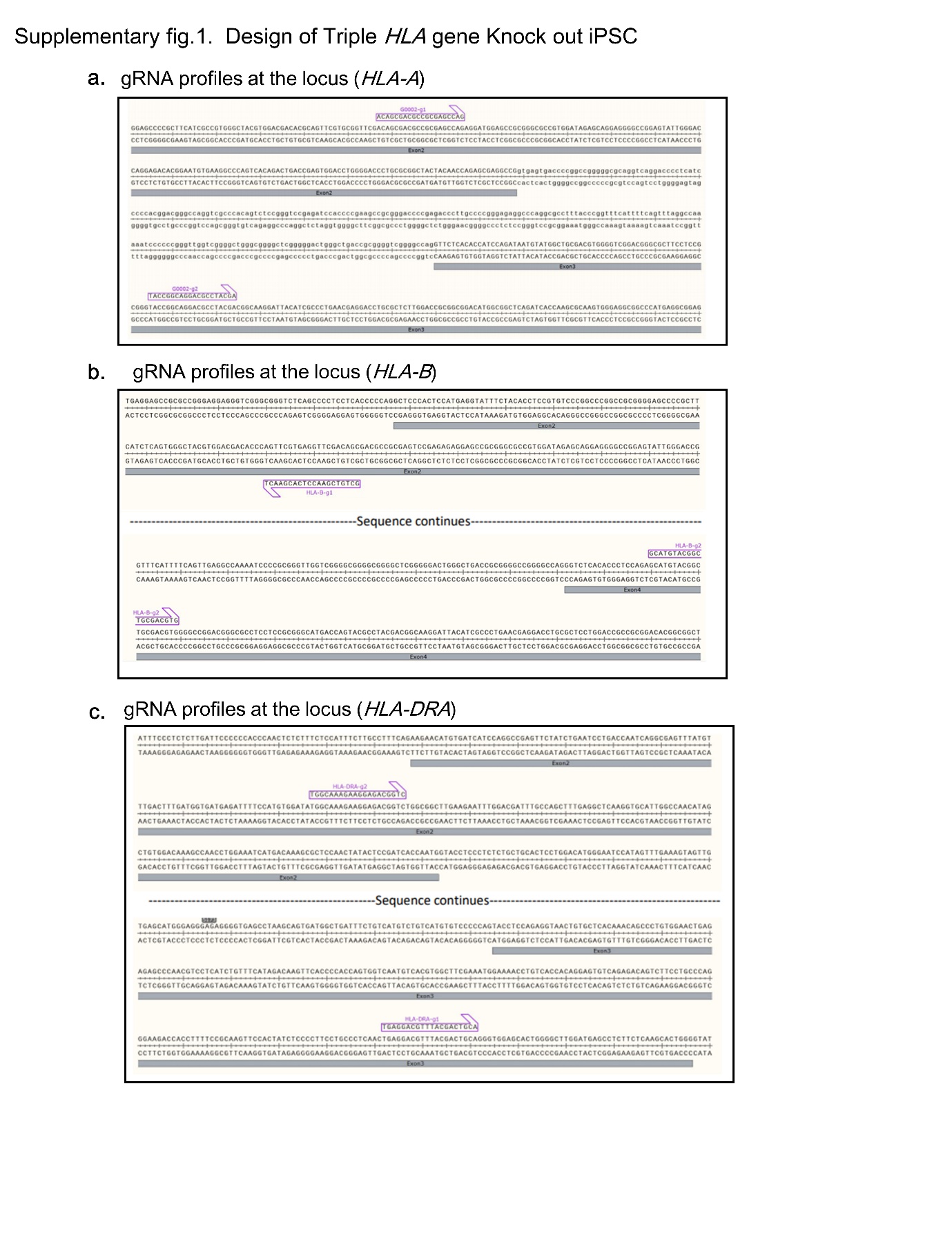
**

**Supplementary Fig. 1.** The profiles of guide RNA (gRNA) candidates targeting human leukocyte antigen *(HLA)-A*, -*B*, and -*DRA* at each locus

**a.** The profiles of two gRNA candidates targeting *HLA-A* at *HLA-A* locus. **b.** The profiles of two gRNA candidates targeting *HLA-B* at *HLA-B* locus. **c.** The profiles of two gRNA candidates targeting *HLA-DRA* at *HLA-DRA* locus.

**Supplementary Fig. 2.**


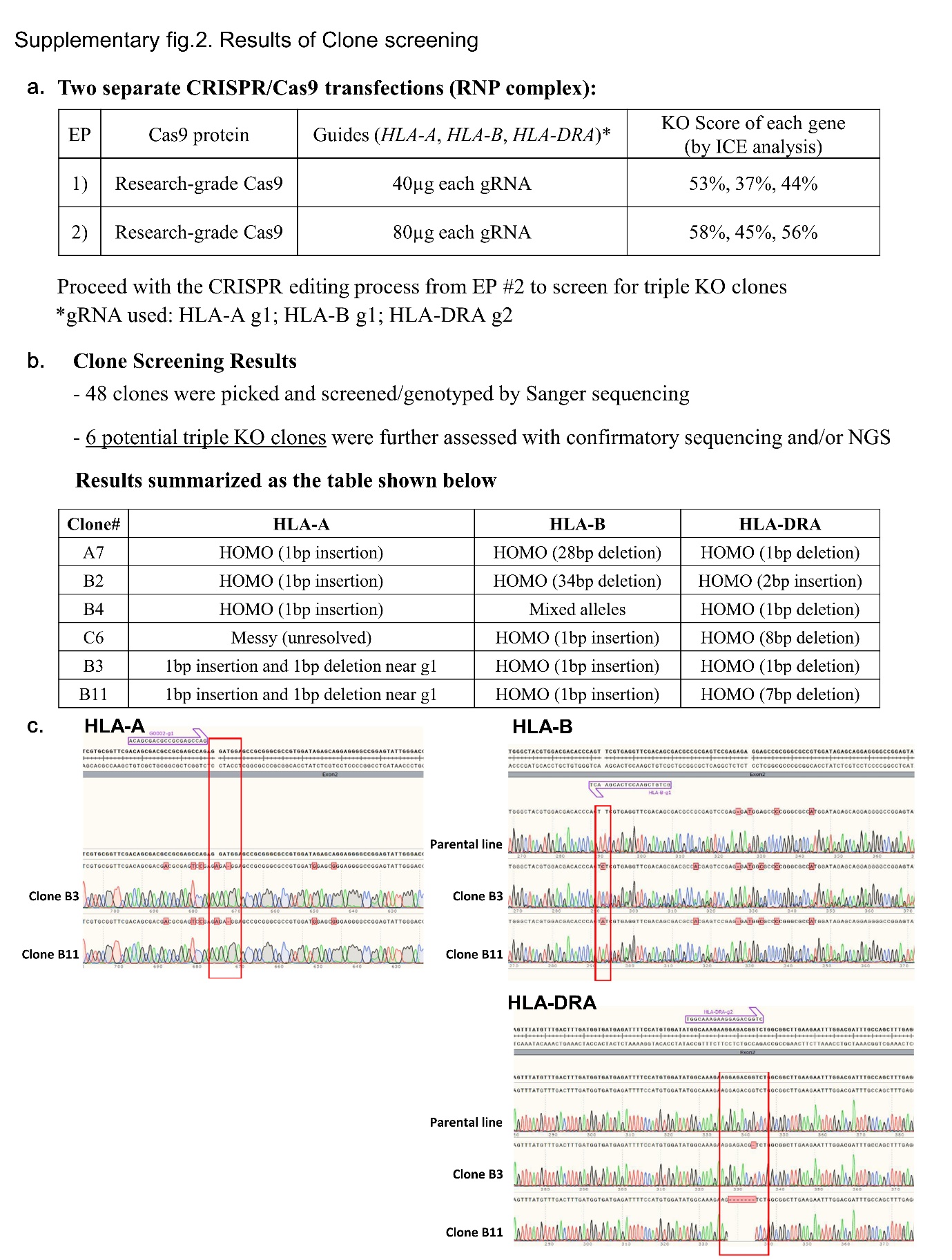


**Supplementary Fig. 2.** Results of clone screening

1. The table representing the conditions of guide RNA (gRNA) and CAS9 ribonucleoprotein (RNP) complex for human leukocyte antigen (*HLA*)-*A*, -*B*, and -*DRA*, alongside the knockout (KO) score of each gene. **b.** The table displays the edited information of engineered clones screened and genotyped through Sanger sequencing. **c.** Sanger sequencing results for gene-edited clone candidates B3 and B11.

**Supplementary Fig. 3.**


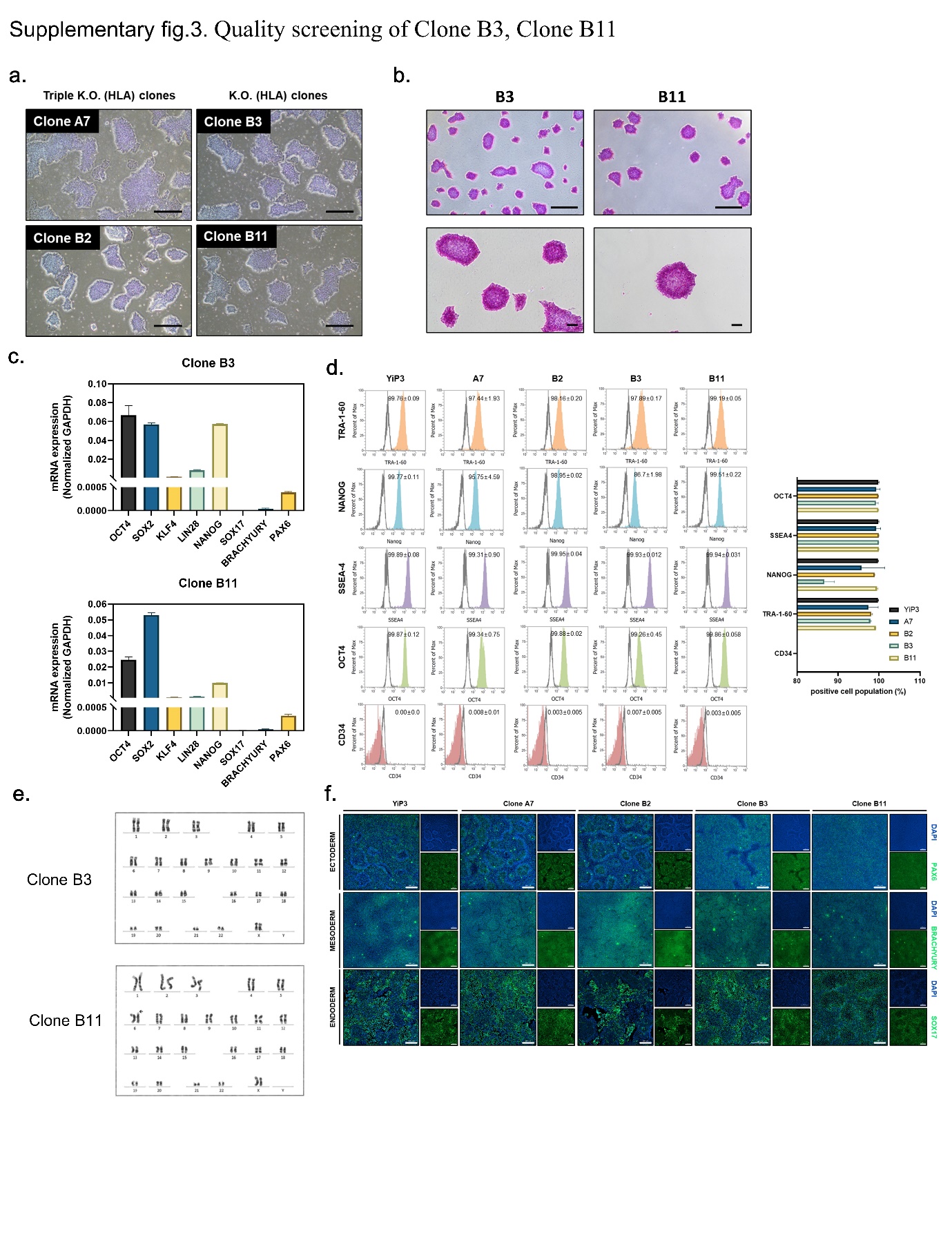


**Supplementary Fig. 3.** Quality screening of clone B3 and B11

**a.** Microscope image of induced pluripotent stem cells (iPSCs), clones A7, B2, B3, and B11. Scale bars, 500 μm. **b.** Alkaline phosphatase (AP) staining image of iPSCs, YiP3, and clones B3 and B11. Scale bars, 500 μm. **c.** The real-time PCR data for the pluripotency markers, such as octamer-binding transcription factor 4 (*OCT4*), SRY (sex-determining region Y)-box 2 (*SOX2*), Krüppel-like factor 4 (*KLF4*), Lin-28 homolog A (*LIN28*), and Nanog homeobox (*NANOG*) and the three germ layer differentiation markers, such as SOX17, BRACHYURY, and paired box 6 (PAX6) in clones B3 and B11. **d.** The flow cytometry data for the pluripotency markers, such as tumor rejection antigen 1-60 (TRA-1-60), NANOG, stage-specific embryonic antigen-1 (SSEA-4), OCT4, and the negative marker CD34 in YiP3 and clones A7, B2, B3, and B11. The bar graph on the right represents the positive cell population for each marker, indicating low expression of NANOG in clone B3. **e.** Karyotyping results of clones B3 and B11. The arrows in the results for clone B11 indicate regions with chromosomal abnormalities. **f.** The immunocytochemistry images demonstrating the expression of markers paired box 6 (PAX6) for ectoderm, BRACHYURY for mesoderm, and SOX17 for endoderm after differentiation into the three germ layers using YiP3 and clones A7, B2, B3, and B11. Scale bars, 200 μm.

**Supplementary Fig. 4.**


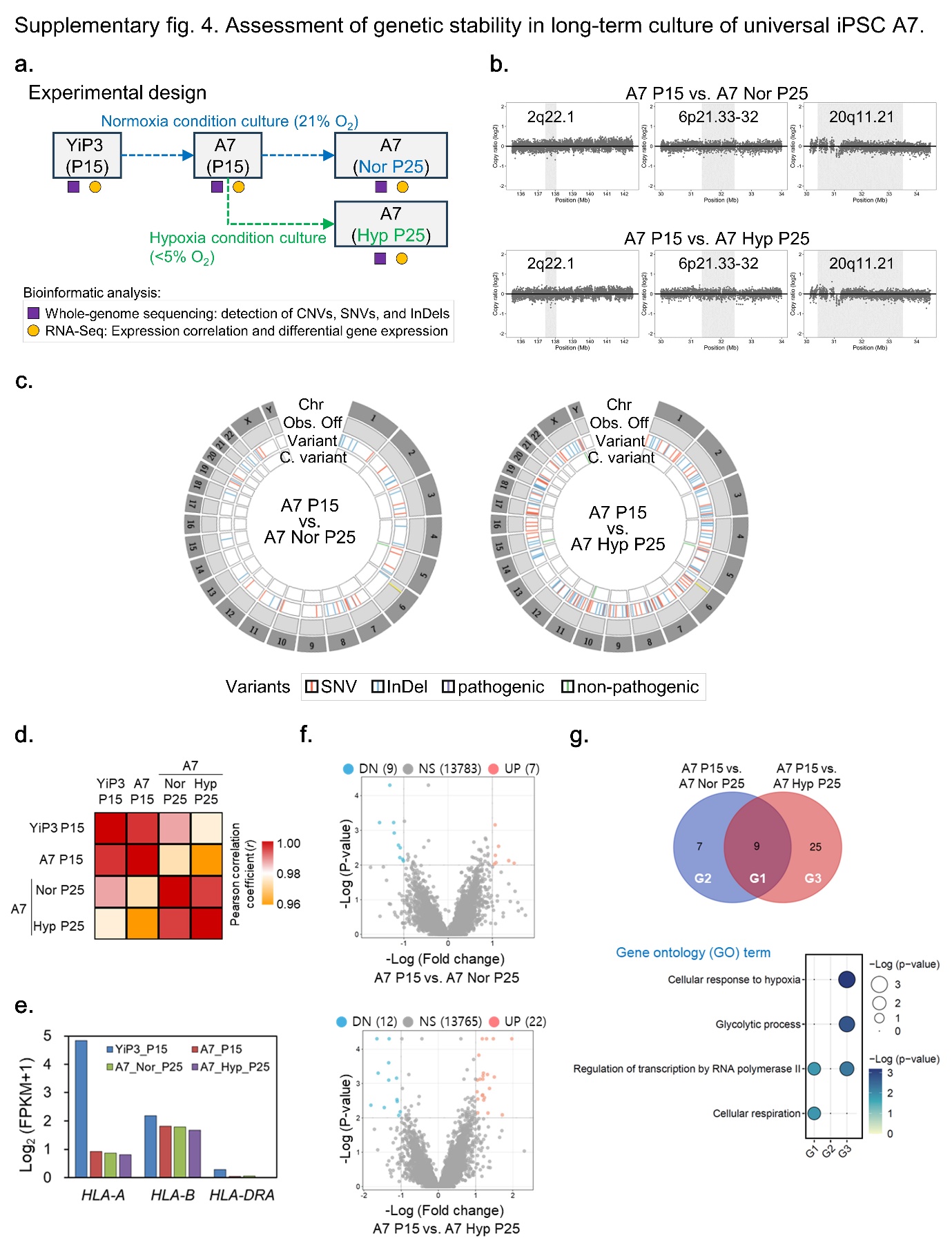


**Supplementary Fig. 4. Assessment of genetic stability in long-term culture of universal iPSC A7**

**a.** Experimental design for assessing the genetic integrity of genome-edited iPSCs during long-term in vitro culture. Genome-edited clone A7 (starting at passage 15) was cultured for an additional 10 passages under both normoxic (21% O_2_) and hypoxic (<5% O_2_) conditions. Whole-genome and RNA sequencing were performed on the cultured samples. **b.** Detection of copy number variations (CNVs) at 2q22.1, 6p21.33-32, and 20q11.21 from whole-genome sequencing data. **c.** Identification of somatic variants, including SNVs and InDels, across all chromosomes. Somatic variants in passage 25 samples were identified by comparison to passage 15 samples. In the Circos plot, the 1st, 2nd, 3rd, and 4th layers represent chromosomes, observed off-target sites, somatic variants (SNVs and InDels), and somatic coding variants (pathogenic and non-pathogenic), respectively. **d.** Correlation heatmap showing transcriptomic similarity between different passages in YiP3 and A7. **e.** Expression of *HLA-A, HLA-B*, and *HLA-DRA* across different passages in YiP3 and A7. **f.** Volcano plot representing differential gene expression. DEGs were identified with a cutoff of *p* < 0.01 and a fold change ≥ 2. **g.** Functional annotation of differentially expressed genes. The Venn diagram in the top panel shows the number of overlapping and unique DEGs between normoxic and hypoxic conditions. In the bottom panel, enrichment of Gene Ontology (GO) terms for DEGs was analyzed using DAVID.

**Supplementary Fig. 5.**


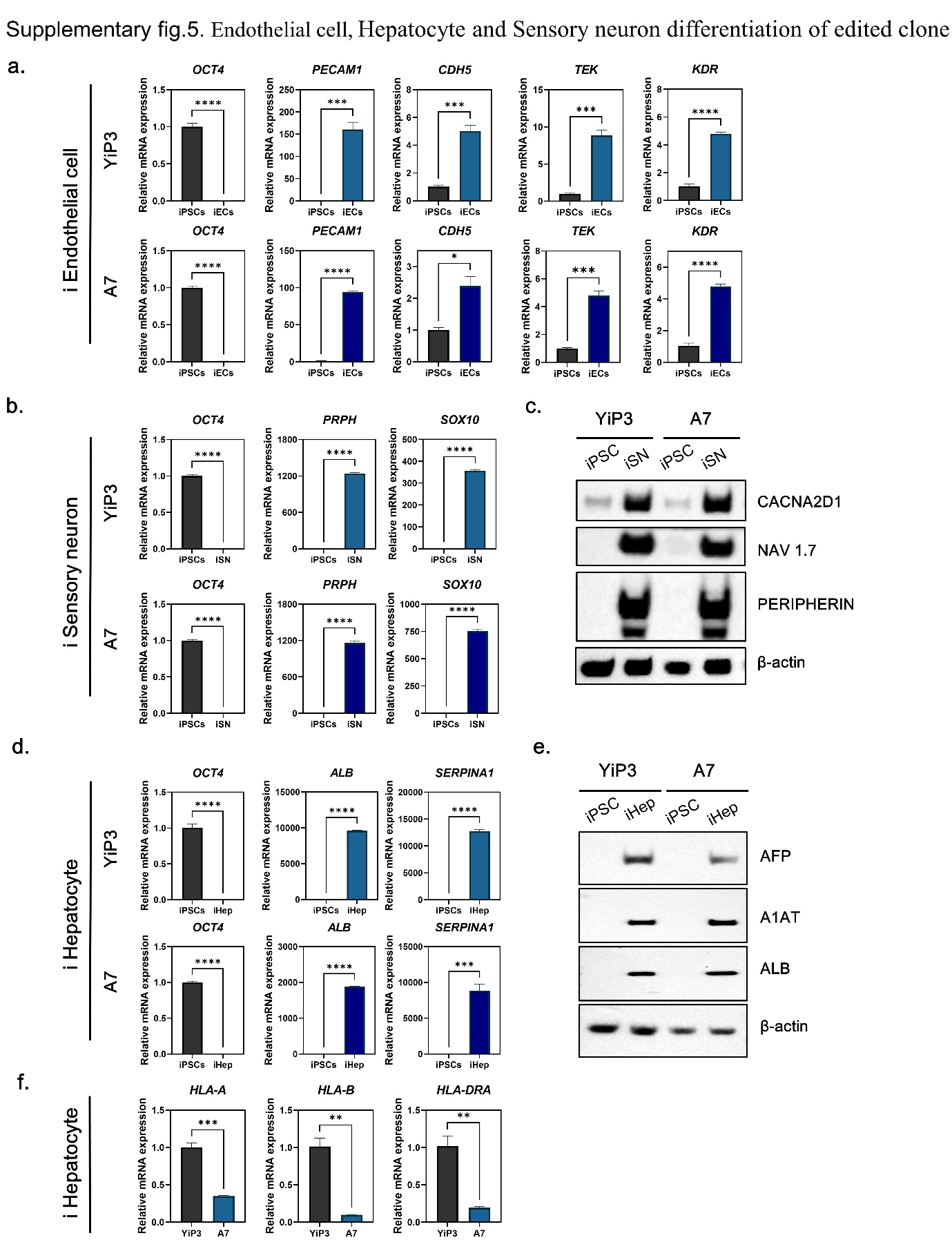


**Supplementary Fig.5. Endothelial cell, Hepatocyte and Sensory neuron differentiation of edited clone**

**a**.The real-time PCR data for the Endothelial cell(iEC) differentiation markers, such as Platelet endothelial cell adhesion molecule (*PECAM*), Cadherin-5 (*CDH5*), *TEK*, Kinase insert domain receptor (*KDR*) and octamer-binding transcription factor 4 (*OCT4*) as pluripotency marker in after endothelial cell differentiation using YiP3 and clones A7. **b.** The real-time PCR data for the sensory neuron (iSN) differentiation markers, such as Peripherin (*PRPH*)*,* SRY-related HMG-box (*SOX10*) as pluripotency marker after sensory neuron differentiation using YiP3 and clones A7. **c**. The western blotting data measured the alterations in the protein expression of Voltage-dependent calcium channel subunit alpha-2/delta-1(CACNA2D1), Sodium voltage-gated channel alpha subunit 9(NAV1.7), PERIPHERIN and β-actin after differentiation into Sensory neurons using YiP3 and clone A7. **d.** The real-time PCR data for the Hepatocyte (iHep) differentiation markers, such as Albumin (*ALB*)*, SERPINA1* and octamer-binding transcription factor 4 (*OCT4*) as pluripotency marker after hepatocyte differentiation using YiP3 and clones A7. **e**. The western blotting data measured the alterations in the protein expression of Alpha-fetoprotein (AFP), Alpha-1 antitrypsin (A1AT), Albumin (ALB) and β-actin after differentiation into Sensory neurons using YiP3 and clone A7. **f.** The real-time PCR data display the mRNA levels of *HLA-A*, *HLA-B*, and *HLA-DRA* after hepatocyte differentiation using YiP3 and clones A7. the graph(**a**,**b**,**d**,**f**) depicts the relative mRNA expression in delta delta cycle threshold (ddCt) values , normalized to glyceraldehyde 3-phosphate dehydrogenase (GAPDH), using iPSCs as the reference value. Statistical significance is indicated by the p-value (Student’s *t* test ) vs. iPSCs **p* < 0.05; ***p* < 0.01; ****p* < 0.001 and *****p* < 0.0001.

**Supplementary Fig. 6.**


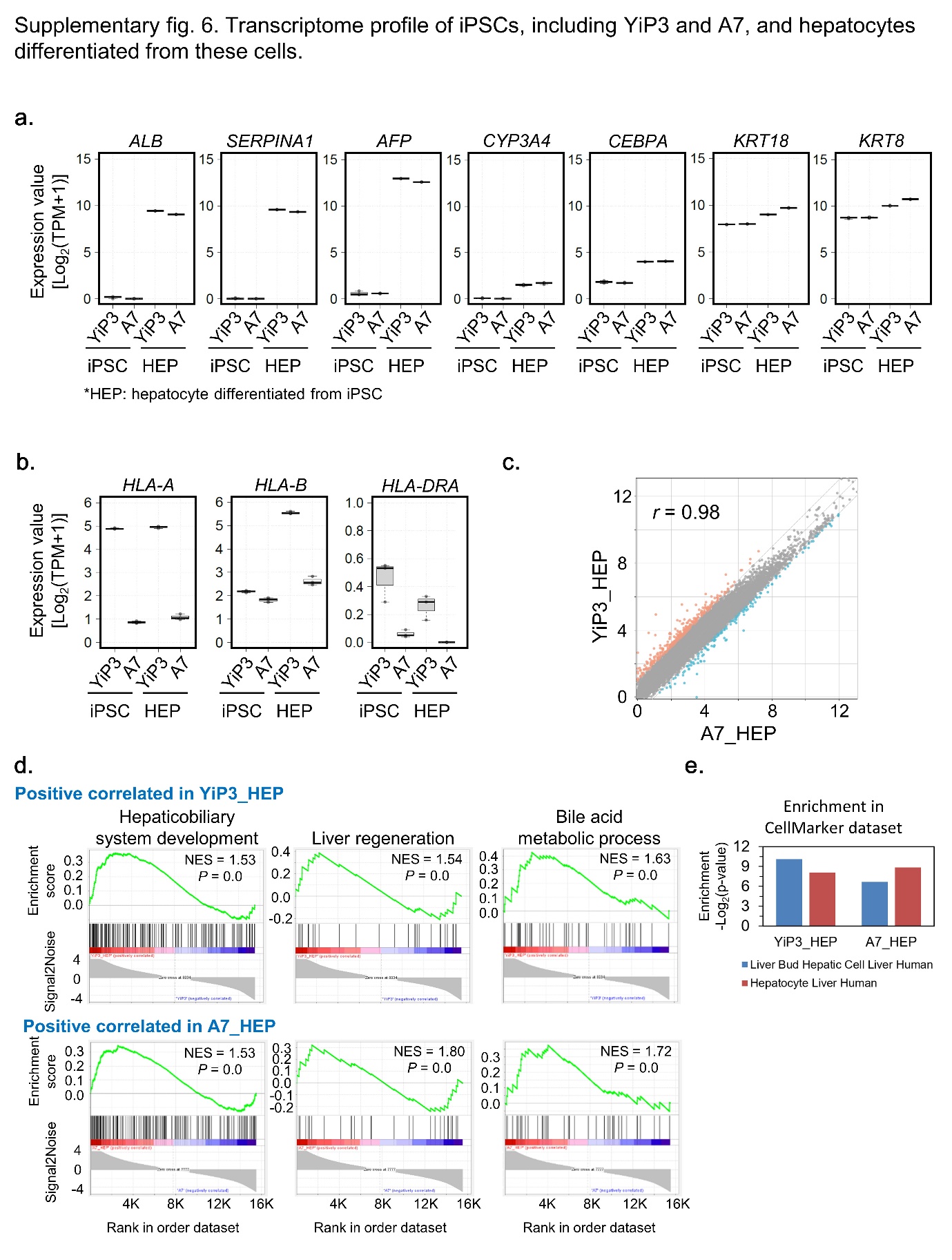


**Supplementary fig. 6. Transcriptome profile of iPSCs, including YiP3 and A7, and hepatocytes differentiated from these cells**

**a**. Expression of hepatocyte markers, including *ALB*, *SERPINA1*, *AFP*, *CYP3A4*, *CEBPA*, *KRT18*, and *KRT8*, in iPSCs (YiP3 and A7) and iPSC-derived hepatocytes (YiP3_HEP and A7_HEP). **b**. Expression of *HLA-A*, *HLA-B*, and *HLA-DRA* in iPSCs and iPSC-derived hepatocytes. **c**. Scatter plot of transcriptome data comparing YiP3_HEP and A7_HEP. **d**. Gene Set Enrichment Analysis (GSEA) plots showing enrichment of hepaticobiliary system development, liver regeneration, and bile acid metabolic processes in YiP3_HEP and A7_HEP. **e**. Enrichment of hepatocyte-specific genes in YiP3_HEP and A7_HEP. Differentially expressed genes in YiP3_HEP and A7_HEP were analyzed by using Enrichr.
